# Molecular Mechanisms Associated with Metabolic Dysfunction: Contributions of Nutritional Genomics

**DOI:** 10.3390/metabo16070501

**Published:** 2026-07-16

**Authors:** Natália Ellen Delmicon, Nathália dos Reis Franco, Giovanna Cavanha Corsi, Roberta Mi Kyong Kim Cho, Helen Cristina Vidal, Marcelo Macedo Rogero

**Affiliations:** 1 Department of Nutrition, School of Public Health, University of Sao Paulo, 715 Dr. Arnaldo Avenue, Sao Paulo 01246-904, SP, Brazil; ndelmicon@gmail.com (N.E.D.); dosnathaliafranco@gmail.com (N.d.R.F.); robkimcho@gmail.com (R.M.K.K.C.); helencvidal@gmail.com (H.C.V.); 2 Department of Food Science and Experimental Nutrition, Faculty of Pharmaceutical Sciences, University of Sao Paulo, 580 Professor Lineu Prestes Avenue, Sao Paulo 05508-000, SP, Brazil; giccorsi@gmail.com

**Keywords:** insulin resistance, inflammation, endotoxemia, polymorphism, single nucleotide, DNA methylation, microRNAs, nutrigenomics

## Abstract

Nutritional genomics has expanded our understanding of how dietary exposures interact with genetic and epigenetic mechanisms involved in metabolic dysfunction. In this context, metabolic dysfunction associated with excessive visceral adiposity arises from a multifaceted interaction between systemic inflammation, insulin resistance, and inter-individual biological susceptibility. Obesity, particularly when driven by diets rich in saturated fatty acids, disrupts intestinal homeostasis, thereby triggering metabolic endotoxemia and contributing to low-grade systemic inflammation and adipose tissue dysfunction. Advances following the Human Genome Project have broadened our understanding of the molecular mechanisms of metabolic diseases, highlighting the role of genetic variability and epigenetic regulation in obesity-related insulin resistance. In nutritional science, the integration of genomics and proteomics has further elucidated how dietary exposures interact with the biological pathways involved in this dysfunction. From a nutrigenomic perspective, this narrative review aims to discuss how genetic variability and diet-related molecular mechanisms contribute to obesity-related metabolic dysfunction, with emphasis on single-nucleotide polymorphisms in key genes, including *FTO*, *MC4R*, *PPAR*, *APOA*, and *FADS*, involved in the regulation of energy homeostasis and insulin secretion. Additionally, we analyze studies on epigenetic mechanisms, including DNA methylation and the action of microRNAs, which act as post-transcriptional regulators sensitive to nutritional and inflammatory stimuli. We also address how dietary patterns, such as the Mediterranean Diet, as well as nutrients and bioactive compounds, can influence epigenetic regulation. We conclude that integrating multiomics data may improve our understanding of the molecular mechanisms underlying metabolic dysfunction and may support the future development of personalized nutritional strategies and molecular biomarkers for obesity and cardiometabolic diseases.

## 1. Introduction

Overweight and obesity constitute a global epidemic with substantial impacts on public health and the economy, affecting countries of all income levels [1,2,3]. According to estimates from the World Health Organization, approximately 2.5 billion adults aged 18 years and older were overweight worldwide in 2022, whereas obesity affected more than 890 million individuals [4]. If current trends continue, the global number of adults living with obesity (body mass index ≥ 30 kg/m^2^) is projected to reach approximately 1.1 billion by 2030 [5].

The dysfunctional expansion of white adipose tissue, especially in the abdominal region, plays a central role in the genesis of systemic low-grade inflammation and insulin resistance (IR), pathophysiological mechanisms underlying chronic non-communicable diseases, including type 2 diabetes (T2D) and cardiovascular diseases (CVD) [6,7].

Although individual biological susceptibility contributes to these alterations, the metabolic phenotype is predominantly influenced by a complex interaction between environmental factors, including diet and physical activity [8,9,10]. Unhealthy dietary patterns and physical inactivity act as triggers for obesity by modulating inflammatory and insulin signaling pathways [8,9]. Among the mediating mechanisms, metabolic endotoxemia stands out, characterized by increased circulating levels of lipopolysaccharides (LPSs), which has been described as an important link between diet and metabolic alterations [9,10,11,12].

Dietary patterns rich in saturated fatty acids (SFAs), as well as refined sugars, induce dysbiosis and increased intestinal permeability, favoring the translocation of LPS into the circulation [9,13]. Once in the bloodstream, LPS activates inflammatory pathways mediated by Toll-like receptors (TLRs), especially TLR4, culminating in the activation of the nuclear factor kappa B (NF-κB) cascade, which sustains systemic low-grade inflammation and impairs insulin signaling in peripheral tissues [6,13,14].

Furthermore, advances driven by the Human Genome Project have broadened the understanding of the molecular basis of these processes, highlighting the role of genetic variability and epigenetic regulation in modulating metabolic responses [15]. In this scenario, the interface between nutritional science and molecular biology has also begun to be explored more systematically through nutritional genomics, which comprises three main sub-areas: nutrigenetics, nutrigenomics, and nutritional epigenomics [15,16,17].

Nutrigenetics investigates how individual genetic variations influence responses to diet, with evidence that single nucleotide polymorphisms (SNPs) in genes related to lipid metabolism, inflammation, and insulin signaling can modulate these effects [18,19,20,21,22]. On the other hand, nutrigenomics studies how direct and indirect interactions between nutrients, bioactive compounds (BCs), or dietary patterns and the genome can influence gene expression [19,23,24]. Complementarily, nutritional epigenomics seeks to understand how nutrients and BCs relate to epigenetic mechanisms that regulate gene expression without altering the deoxyribonucleic acid (DNA) sequence, with emphasis on DNA methylation and non-coding ribonucleic acid, especially microRNAs (miRNAs) [15,16,19,25,26]. These epigenetic mechanisms act as dynamic mediators at the interface between nutritional and inflammatory stimuli, regulating gene expression in different tissues and impacting metabolic pathways involved in the pathogenesis of non-communicable diseases [13,16,27,28].

Although several reviews have explored the relationship between nutritional genetics and metabolic diseases, a gap remains in the literature regarding an integrative synthesis that simultaneously encompasses nutrigenetics, nutrigenomics, and nutritional epigenomics in the context of metabolic dysfunction affecting glucose, lipid, and inflammatory pathways. Existing reviews have generally addressed only individual components of this framework. Some focus primarily on nutrigenetics [29] or nutrigenomics [30], whereas others are restricted to mechanisms underlying glucose metabolism and T2D [30,31] or inflammation [15]. Additional reviews have emphasized precision nutrition for weight management [32] or have focused predominantly on miRNAs without providing a comprehensive discussion of DNA methylation [15]. This fragmented perspective limits a comprehensive understanding of the molecular mechanisms underlying the interplay between diet, genetic susceptibility, and metabolic dysfunction.

Given the multifactorial nature of obesity-related metabolic dysfunction, an integrated discussion of these complementary perspectives can provide a broader understanding of the molecular mechanisms linking dietary exposure to disease development. Accordingly, this narrative review synthesizes and integrates current evidence on diet-induced metabolic endotoxemia, LPS–TLR4/NF-κB-mediated inflammation, insulin resistance (IR), genetic susceptibility—with particular emphasis on genetic polymorphisms, nutrigenomics, and nutritional epigenomics—including the major epigenetic mechanisms, namely DNA methylation and miRNAs, to provide a comprehensive perspective on the molecular pathways linking diet to obesity-related metabolic dysfunction.

## 2. Metabolic Inflammation and Insulin Resistance

### 2.1. Metabolic Endotoxemia: The Role of LPS in the Liver, Adipose Tissue, and Macrophages

Inflammation involves the interaction of multiple cell types, particularly those of the immune system. It is initiated when immune cells recognize pathogen-associated molecular patterns (PAMPs) or damage-associated molecular patterns (DAMPs) through their pattern recognition receptors (PRRs), especially TLRs [12]. Activated immune cells release pro-inflammatory cytokines, such as tumor necrosis factor alpha (TNF-α) and interleukin-6 (IL-6), leading to increased oxidative stress, vasodilation, and vascular permeability [12,33]. Inflammation serves as a physiological role when it aims at pathogen clearance, tissue repair, and the restoration of homeostasis. A balanced and well-regulated inflammatory response is essential for survival and tissue recovery. However, inflammation may acquire a pathophysiological state when tolerance is lost or when signaling becomes dysregulated, leading to tissue damage and disease development, such as metabolic dysfunction-associated steatotic liver disease (MASLD), CVD, metabolic syndrome (MetS), and T2D [12,33,34].

**Figure 1:**
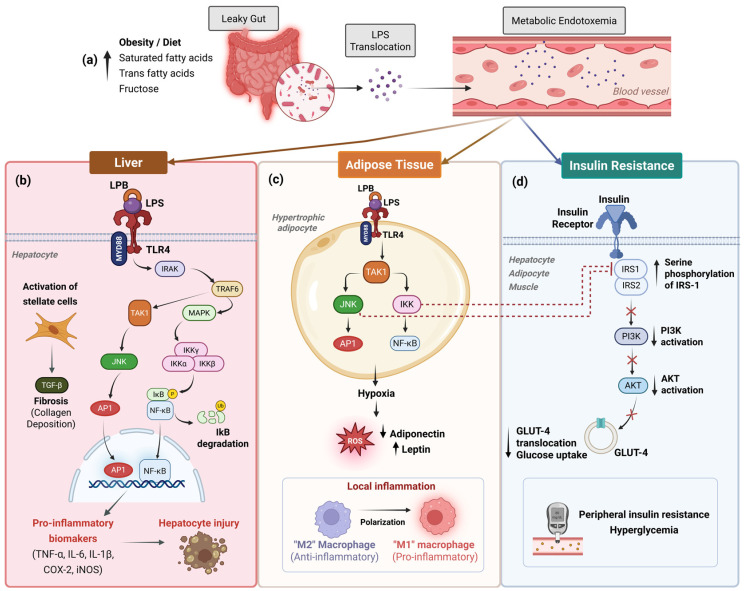
Molecular mechanisms of metabolic endotoxemia and its role in systemic inflammation and insulin resistance. (**a**) Obesity and diets high in saturated fatty acids, trans fatty acids, and fructose promote gut dysbiosis and heightened intestinal permeability, facilitating the translocation of gut-derived lipopolysaccharide (LPS) into the systemic circulation. (**b**) In the liver, LPS activates Toll-like receptor 4 (TLR4), triggering the MyD88-dependent pathway (IRAK–TRAF6–TAK1) and leading to the activation of nuclear factor kappa B (NF-κB) and activator protein 1 (AP-1). This process enhances the production of pro-inflammatory cytokines, including TNF-α, IL-6, IL-1β, promotes hepatocellular injury, and activates hepatic stellate cells. (**c**) In WAT, TLR4 activation by LPS induces NF-κB and AP-1 pathways, promoting a chronic, low-intensity inflammatory response. Adipocyte hypertrophy favors hypoxia and increases reactive oxygen species (ROS), contributing to local inflammation. (**d**) In metabolic tissues (liver, WAT, and skeletal muscle), the activation of serine kinases such as c-Jun N-terminal kinase (JNK) and IκB kinase (IKK) promotes serine phosphorylation of IRS-1/2. This inhibits PI3K/Akt signaling and blunts glucose transporter type 4 (GLUT4) translocation, thereby compromising glucose uptake and exacerbating hyperglycemia and insulin resistance. Abbreviations: Akt, protein kinase B; IL-1β, interleukin 1 beta; IL-6, interleukin 6; iNOS, inducible nitric oxide synthase; IRAK, interleukin-1 receptor-associated kinase; IRS, insulin receptor substrate; MAPK, mitogen-activated protein kinase; MyD88, myeloid differentiation primary response 88; PI3K, phosphoinositide 3-kinase; ROS, reactive oxygen species; TAK1, transforming growth factor-β-activated kinase 1; TGF-β, transforming growth factor beta; TNF-α, tumor necrosis factor alpha; TRAF6, TNF receptor-associated factor 6. Source: Created by the authors using BioRender. Corsi, G. (2026) https://BioRender.com/u1sydig.

A primary driver of the systemic inflammatory response associated with obesity is LPS, a structural component of the Gram-negative bacterial cell wall [11,33]. Obesity and deleterious dietary patterns can alter gut microbiota composition and heighten intestinal permeability, a phenomenon frequently termed “leaky gut” [11,12]. Compromise of the intestinal barrier triggers metabolic endotoxemia, a condition characterized by a chronic, two-to-three-fold elevation in gut-derived plasma LPS levels. This sustained increase induces low-grade, chronic systemic inflammation, consequently activating pro-inflammatory signaling cascades that may exacerbate peripheral IR, hyperglycemia, hepatic injury, and the development of cardiometabolic diseases (Figure 1a) [11,12,35].

LPS crosses the intestinal barrier and reaches the portal or systemic circulation through transcellular and paracellular pathways [11,35]. Within the cellular environment, LPS is recognized by PRRs, particularly TLR4, and this interaction initiates a complex cascade of inflammatory and immune responses. TLR4 is expressed on the membrane of various cell types, including both immune cells—such as monocytes, macrophages, and dendritic cells—and non-immune cells, such as endothelial cells, adipocytes, and hepatocytes [33,36].

Initially, LPS binds to lipopolysaccharide-binding protein (LBP). The LBP–LPS complex is then transferred to and recognized by cluster of differentiation 14 (CD14), which may be membrane-bound or soluble. Following LPS binding, CD14 presents this PAMP to TLR4. LPS binds to TLR4 with the assistance of myeloid differentiation factor 2 (MD-2), with the ligand positioned between TLR4 and MD-2 [11,33,37].

LPS binding to TLR4 promotes receptor dimerization, inducing conformational changes that favor the recruitment of additional signaling molecules. The Toll/Interleukin-1 Receptor (TIR) domain of TLR4 interacts with the TIR domain-containing adaptor protein (TIRAP) and the cytoplasmic adaptor protein MyD88 [33,37,38,39]. This interaction activates members of the IL-1 receptor–associated kinase (IRAK) family and recruits IRAK4, IRAK1, and IRAK2 to form a multiprotein complex. Phosphorylation of IRAK promotes its association with tumor necrosis factor receptor–associated factor 6 (TRAF6) [37,38,39]. TRAF6, together with the E2 ubiquitin-conjugating enzyme complex composed of Ubc13 and Uev1A, catalyzes the formation of K63-linked polyubiquitin chains, which in turn activate transforming growth factor-β–activated kinase 1 (TAK1) and TAK-binding proteins (TABs), TAB1, TAB2, and TAB3 [33,37,38,39]. From the TABs-TAK1 complex, mitogen-activated protein kinase (MAPK) and the IκB kinase (IKK) complex are activated. IKK consists of two catalytic subunits (IKK-α and IKK-β) and a regulatory subunit designated NEMO, also called IKKγ. IKK-α and IKK-β induce phosphorylation of inhibitor of κB (IκB)-α, leading to its polyubiquitination and subsequent degradation by the 26S proteasome. This process allows the p50/p65 heterodimer (nuclear factor kappa B, NF-κB) to translocate to the nucleus [33,37,38,39].

In parallel, TAK1 can also activate the activator protein-1 (AP-1) inflammatory pathway. AP-1 is a dimeric transcription factor composed primarily of Jun and Fos family members, although it may also include Jun-dimerizing partners, the Musculoaponeurotic fibrosarcoma family, and the Activating transcription factor family [40,41]. TAK1 phosphorylates c-Jun N-terminal kinase (JNK), which in turn activates AP-1 [39].

Activated NF-κB and AP-1 transcription factors translocate to the nucleus and bind to their specific DNA sequences. This binding induces the transcription of numerous genes involved in the inflammatory response, including those encoding pro-inflammatory cytokines (TNF-α, IL-1β, IL-6), cell adhesion molecules such as intercellular adhesion molecule-1 (VCAM-1) and vascular cell adhesion molecule-1 (ICAM-1), chemokines (CCL2, CCL3, CCL4, CCL5, CXCL1, CXCL2), cyclooxygenase-2 (COX-2), and inducible nitric oxide synthase (iNOS). Furthermore, in macrophages, activated NF-κB promotes M1 polarization [35,37,38,39].

Via the portal circulation, LPS reaches the liver, where it is recognized by hepatocytes, hepatic stellate cells, and resident macrophages (Kupffer cells) and is subsequently excreted into the bile duct [11,12,35]. Impaired hepatic capacity to metabolize and excrete LPS into bile, as observed, for example, in obesity-associated hepatic lipid accumulation, exacerbates endotoxemia and sustains systemic inflammation [11,12].

TLR4 expression is low in Kupffer cells, hepatocytes, and hepatic stellate cells under physiological conditions in a healthy liver. However, inflammation upregulates this receptor, which in turn sustains the inflammatory response. Increased hepatic TLR4 expression, together with the release of inflammatory mediators, such as TNF-α, and oxidative stress, contributes to liver injury and represents a central pathway in the pathogenesis of alcoholic liver disease and MASLD. This upregulation is also associated with the activation of fibrogenic cells and the progression of fibrosis [11,35]. Activation of hepatic stellate cells can lead to the overexpression of fibrogenic genes, including transforming growth factor-β (TGF-β). Activated TGF-β promotes collagen deposition and induces the differentiation of fibroblasts into myofibroblasts, the principal source of extracellular matrix production. Although collagen is essential for tissue repair, its excessive production and accumulation are central to pathological hepatic fibrosis (Figure 1b) [11,35,42,43].

LPS also activates TLR4 in adipocytes [36,44]. In WAT, inflammation and/or excess body fat induces adipocyte hypertrophy, which is characterized by cell enlargement and increased intracellular lipid storage. This expansion generates areas of low oxygen (O_2_) pressure, a phenomenon known as hypoxia, which enhances mitochondrial production of reactive oxygen species (ROS) and induces compensatory angiogenesis. Changes in oxygen levels are primarily regulated by hypoxia-inducible factors (HIFs). Under normoxic conditions, oxygen sensors known as prolyl hydroxylase domain enzymes (PHDs) and factor inhibiting HIF (FIH) are activated, promoting proteolytic degradation and maintaining low HIF-1α activity. However, under hypoxic conditions, the activity of PHDs and FIH is inhibited, leading to HIF-1α stabilization and accumulation. Stabilized HIF-1α translocates to the nucleus, where it forms a transcriptional complex with HIF-1β and activates the expression of genes involved in angiogenesis, such as vascular endothelial growth factor and erythropoietin, as well as the production of inflammatory cytokines such as IL-1β [45,46]. Intracellular lipid overload in adipocytes reduces adiponectin expression and secretion, elevates leptin levels, and triggers local inflammation. This process promotes pro-inflammatory macrophage infiltration and M1-phenotype polarization, thereby reinforcing systemic inflammatory cascades (Figure 1c) [12,44,47,48].

WAT can be classified into visceral adipose tissue (VAT) and subcutaneous adipose tissue (SAT). SAT is located beneath the skin, whereas VAT surrounds internal organs within the abdominal cavity and includes omental, mesenteric, retroperitoneal, gonadal, and pericardial WAT. VAT exhibits a more pronounced pro-inflammatory profile, characterized by greater metabolic activity, increased infiltration of M1-phenotype macrophages, elevated secretion of pro-inflammatory cytokines, higher lipolytic capacity, and continuous release of free fatty acids (FFAs) [34,49]. Consequently, VAT contributes to systemic inflammation and is associated with metabolic dysregulation and disorders such as IR, T2D, and CVD [34,49,50].

### 2.2. Nutrients, Inflammatory Response, and Insulin Resistance

**Figure 2:**
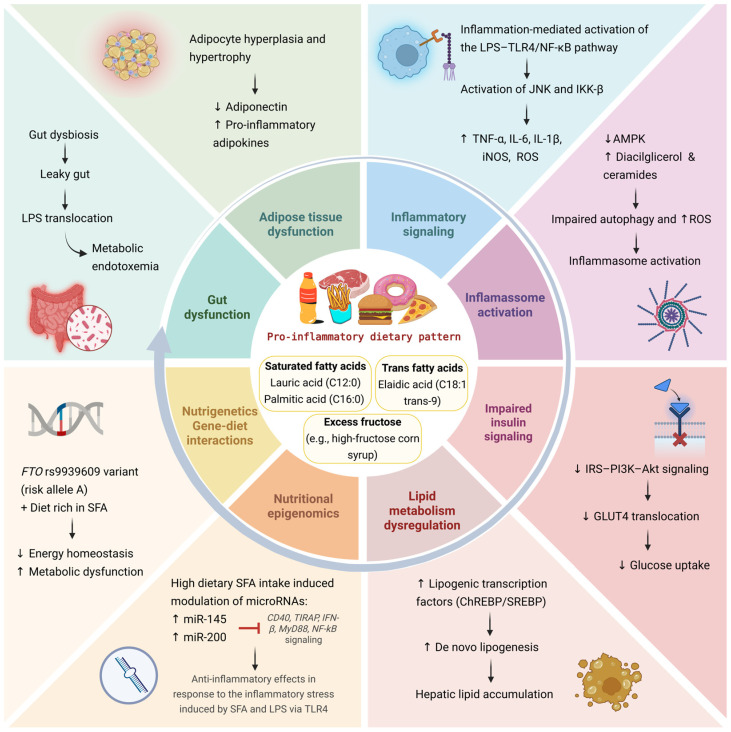
Integrated molecular mechanisms linking pro-inflammatory dietary components, nutritional genomics, and metabolic dysfunction. A dietary pattern characterized by high intakes of saturated fatty acids (primarily lauric acid (C12:0) and palmitic acid (C16:0)), industrial trans fatty acids (mainly elaidic acid (C18:1 trans-9)), and excessive fructose (e.g., high-fructose corn syrup) promotes metabolic dysfunction through multiple interconnected mechanisms. These include gut dysbiosis and metabolic endotoxemia, adipose tissue dysfunction, activation of inflammatory signaling pathways, inflammasome activation, impaired insulin signaling, dysregulated lipid metabolism, gene–diet interactions, and nutritional epigenomics mechanisms. Together, these alterations contribute to chronic low-grade inflammation, insulin resistance, impaired glucose uptake, ectopic lipid accumulation, and metabolic dysfunction. Abbreviations: Akt, protein kinase B; AMPK, AMP-activated protein kinase; CD40, cluster of differentiation 40; ChREBP, carbohydrate-responsive element-binding protein; FTO, fat mass and obesity-associated gene; GLUT4, glucose transporter type 4; IFN-β, interferon beta; IKK-β, IκB kinase beta; IL-1β, interleukin-1 beta; IL-6, interleukin-6; iNOS, inducible nitric oxide synthase; IRS, insulin receptor substrate; JNK, c-Jun N-terminal kinase; LPS, lipopolysaccharide; MyD88, myeloid differentiation primary response protein 88; NF-κB, nuclear factor kappa B; PI3K, phosphoinositide 3-kinase; ROS, reactive oxygen species; SFA, saturated fatty acids; SREBP, sterol regulatory element-binding protein; TIRAP, Toll/interleukin-1 receptor domain-containing adaptor protein; TLR4, Toll-like receptor 4; TNF-α, tumor necrosis factor alpha. **Symbols:** ↑, increased level or activity; ↓, decreased level or activity; ⊣, inhibition. Source: Created in BioRender. Corsi, G. (2026) https://BioRender.com/vyijqqj.

Obesity, inflammation and inadequate eating patterns (especially those rich in SFAs, trans fatty acids, and excessive fructose intake) are strongly associated with IR, leading to reduced glucose uptake, hyperglycemia, and, consequently, T2D (Figure 2) [13,51,52].

Insulin is a key hormone produced by β cells of the pancreatic islets of Langerhans. In skeletal muscle and WAT, insulin regulates glucose and lipid homeostasis by promoting glucose uptake and lipogenesis and suppressing gluconeogenesis and glycogenolysis [13,53]. Insulin action begins with its binding to the extracellular α-subunit of the insulin receptor. This binding leads to the phosphorylation of insulin receptor substrates (IRS)-1 and IRS-2 at tyrosine residues, which subsequently results in the recruitment of phosphatidylinositol 3-kinase (PI3K). PI3K phosphorylates phosphatidylinositol-4,5-bisphosphate (PIP2), converting it into phosphatidylinositol-3,4,5-trisphosphate (PIP3). This, in turn, recruits and activates pyruvate dehydrogenase kinase 1 (PDK1), which subsequently phosphorylates protein kinase B (PKB), also known as Akt. In skeletal muscle and WAT, PKB/Akt promotes the translocation of glucose transporter type 4 (GLUT4) to the plasma membrane, thereby facilitating cellular glucose uptake [13,53,54,55].

A central mechanism of SFA-induced IR involves disruption of IRS signaling. Diets rich in SFAs, primarily lauric acid (C12:0) and palmitic acid (C16:0), are associated with increased inflammation and accumulation of lipid intermediates, including diacylglycerol (DAG) and ceramides [51,52,55]. Through inflammatory signaling and DAG and ceramide accumulation, SFAs activate IKK-β and JNK, which promote the phosphorylation of IRS at serine/threonine residues. This modification reduces normal tyrosine phosphorylation of IRS and impairs proper insulin signaling (Figure 1d) [13,14,51,55]. In addition, inflammatory mediators such as TNF-α and IL-6 increase the expression of suppressors of cytokine signaling (SOCS) proteins, which act as competitive inhibitors of IRS tyrosine phosphorylation and promote ubiquitination and proteasomal degradation of IRS, further contributing to IR [14,51,53].

Another known mechanism of SFAs, especially palmitic acid, is the activation of inflammasomes, which contributes to chronic low-grade metabolic inflammation and IR [56,57,58,59,60]. SFAs inhibit the phosphorylation and activation of AMP-activated protein kinase (AMPK), resulting in impaired autophagy and accumulation of ROS, thereby promoting inflammasome activation [57,60]. In addition, SFAs promote the synthesis of DAG and ceramides, which are associated with increased inflammasome activation [60]. The NLRP3 inflammasome (NOD-like receptor protein 3) is a multiprotein complex involved in the regulation of innate immunity and inflammation [56,58]. This complex consists of a sensor (NLRP3), an adaptor (apoptosis-associated speck-like protein—ASC) and an effector (caspase-1) [56,58,61]. Inflammasome activation promotes the cleavage of procaspase-1 into active caspase-1, a protease that catalyzes the conversion of pro-IL-1β and pro-IL-18 into their active forms, increasing the secretion of these inflammatory cytokines [60,62].

Industrial trans fatty acids, notably elaidic acid (C18:1 trans-9), are potent mediators of metabolic dysfunction, linked to impaired insulin sensitivity and a heightened risk of T2D [52,63,64,65,66]. Beyond their association with systemic inflammation, trans fatty acids exert deleterious effects on cellular homeostasis by impairing endothelial function and promoting intestinal dysbiosis. At the molecular level, trans fatty acid intake exacerbates endoplasmic reticulum stress and augments the production of ROS [63,67,68]. Furthermore, these fatty acids disrupt adipocyte physiology by altering adipokine secretion profiles and interfering with adipocyte differentiation, which facilitates visceral fat accumulation and tissue dysfunction. Notably, trans fatty acids directly stimulate pro-inflammatory signaling through the activation of the NF-κB pathway, thereby perpetuating a state of chronic, low-grade inflammation [63,67].

Excessive fructose intake (such as high-fructose corn syrup) disrupts metabolic homeostasis, leading to hyperglycemia and an increased risk of IR [69,70,71]. After absorption, fructose enters the bloodstream and is transported to the liver, where it is primarily taken up by hepatocytes. In the liver, fructose can be metabolized and converted into glucose and lactate or enter the de novo lipogenesis pathway [69]. Fructose uptake into cells is an insulin-independent process. This pathway allows rapid and unregulated fructose uptake by hepatocytes, exacerbating metabolic stress and increasing the production of lactate, triglycerides, and ROS within cells [69]. The main mechanism linking excessive fructose intake to de novo lipogenesis is the activation of lipogenic transcription factors, such as carbohydrate responsive element-binding protein (ChREBP) and sterol-responsive element-binding protein (SREBP). Once activated, ChREBP and SREBP stimulate the expression of fatty acid synthase and acetyl-CoA carboxylase (ACC), leading to increased fatty acid synthesis, excess liver fat deposition and MASLD [69,72,73]. Additionally, fructose consumption can induce gut dysbiosis and alter bacterial metabolites. This effect is associated with decreased expression of intestinal tight junction proteins, such as zonula occludens-1, leading to “leaky gut” and facilitating the translocation of microbial toxic compounds, such as LPS, into the bloodstream, thereby triggering an inflammatory response [71,72,74].

## 3. Genetic and Epigenetic Determinants of Insulin Resistance and Metabolic Inflammation

### 3.1. Genetic and Epigenetics Mechanisms

The development of metabolic dysfunction results from a complex interaction between genetic predisposition and environmental factors [75,76]. Genetic variations, such as nucleotide substitutions, can alter how the DNA sequences are interpreted by cellular machinery. This can lead to the synthesis of altered proteins and, consequently, impair their optimal protein function [77].

However, in the context of disease development, genetic predisposition alone is not deterministic. The phenotype is strongly influenced by environmental factors, such as diet and physical inactivity, through interactions with epigenetic mechanisms that transmit exogenous signals without altering the DNA base sequence [75,76,78]. Histone modifications, DNA methylation, and the action of non-coding RNAs constitute the primary epigenetic regulatory mechanisms. These mechanisms promote changes at the transcriptional and/or post-transcriptional levels, thereby modulating gene expression, and they are potentially reversible [76,79]. In this context, the pathophysiology of metabolic diseases results from the dynamic integration of genetic, epigenetic, and environmental factors.

### 3.2. Genetic Variability and Metabolic Dysfunction

Obesity is a chronic and multifactorial disease influenced by the interaction between environmental factors, lifestyle, and individual genetic susceptibility. Evidence from longitudinal analyses indicates that obesity-related clinical severity, assessed beyond body mass index alone, is associated with a higher all-cause and cause-specific mortality risk. Furthermore, gene-diet studies suggest that genetic variability may modulate individual susceptibility to obesity and metabolic responses to dietary interventions [80,81].

In the context of nutrigenetics, genetic variations in the nucleotide sequence of DNA, such as for SNPs, can modulate metabolic pathways in response to the intake of nutrients, bioactive food compounds, and dietary patterns [82]. Recent evidence suggests that SNPs can influence genes involved in energy homeostasis, insulin signaling, and inflammatory response [29,83]. Furthermore, genome-wide association studies (GWAS) conducted in diverse populations have identified numerous genetic variations and candidate genes associated with disease, which has advanced the understanding of individual susceptibility to the development of metabolic disorders [84].

Extensive genetic research in recent decades has implicated SNPs in key genes in the development of metabolic diseases. A primary example is the rs7903146 SNP in the transcription factor 7-like 2 (*TCF7L2*) gene, which is strongly associated with the incidence of T2D by modulating insulin sensitivity, glycemic levels, and inflammatory biomarkers. Nevertheless, recent research on other *TCF7L2* variations has demonstrated pleiotropic effects on human health [85].

*TCF7L2*, a gene on chromosome 10, can be expressed in various tissues, such as the islets of Langerhans and the liver, and is a transcriptional regulator of the canonical Wnt signaling pathway by which pancreatic β-cell proliferation is controlled [86,87]. Recent experimental evidence indicates that impaired *TCF7L2* activity may contribute to pancreatic β-cell dysfunction by promoting β-cell dedifferentiation and reducing insulin secretion. In a murine pancreatic β-cell line, *TCF7L2* silencing increased the expression of hairy and enhancer of split 1, reduced the expression of forkhead box O1, and decreased insulin secretion—effects that were attenuated after inhibition of extracellular signal-regulated kinase phosphorylation. In mice with β-cell-specific *TCF7L2* deficiency, *TCF7L2* deletion impaired glucose tolerance and was accompanied by increased activation of the extracellular signal-regulated kinase/mitogen-activated protein kinase signaling pathway and changes consistent with β-cell dedifferentiation. These findings support a role for *TCF7L2* dysfunction in impaired insulin secretion and altered glucose homeostasis [88].

The SNP rs7903146 (C > T) of *TCF7L2*, an intronic variant, has been indicated as a potential established genetic biomarker to predict the risk for T2D in distinct ethnic and geographic groups, as observed in a systematic review that presented a significant association of the presence of the T allele, in TT or CT genotypes, with an elevated risk for the development of T2D in studies with populations of African, South Asian, and European descent [89]. The frequency of the dominant TT genotype observed in the Chinese population and the frequency of the T allele in Latin populations, mainly of mixed and Brazilian descent, were significantly associated with a higher risk of T2D [90].

In a meta-analysis of GWAS conducted with approximately 2.5 million individuals, of which 39.7% corresponded to the population of non-European ancestry and diverse ancestries, such as East and South Asian, Hispanic, and African, 1289 independent genetic signals, i.e., index SNPs, which allow fine mapping to detect the SNP responsible for the biological alteration associated with the risk of developing T2D, were identified in 611 genetic loci, of which 145 were discovered [91]. Furthermore, this study not only identified new genes but also pointed to the *TCF7L2* gene as one of the example loci, demonstrating robust evidence of association already presented in studies related to SNP, IR and T2D, and corroborating a direct association of this gene with pancreatic β-cell dysfunction and insulin secretion that were significantly observed in populations of East Asian ancestry compared to those of Europe and Africa, which may justify the incidence of T2D in the Indian population despite the eutrophic body mass index [91].

A study conducted with participants of European descent, which addressed the interaction between genetic variants and BMI in association with metabolic MASLD, suggests a protective effect against diabetes and MASLD in individuals with high BMI in the presence of the G allele of the rs34783010 SNP of the gastric inhibitory polypeptide receptor (*GIPR*) gene, whose essential role is related to the control of systemic energy metabolism through signaling in the central nervous system. On the other hand, the presence of the T allele demonstrated an association with a greater propensity for the development of T2D, elevated HbA1c and triglycerides, and reduced BMI [92].

The *SLC30A8* gene encodes the protein that acts in the transport of zinc ions (ZnT8) to the granules of the insulin secretory pathway in pancreatic β cells—essential for insulin storage—and the SNP rs13266634 is associated with a protective role in susceptibility to T2D in individuals with the TT genotype in the Bangladeshi population [93]. However, a cross-sectional study presented new associations between *SLC30A8* gene SNPs and metabolic and lipid profiles that go beyond the knowledge of the influence of the genetic variant on the physiological relevance of T2D, showing that CC genotypes of SNP rs13266634 and AA genotypes of SNP rs11558471 were associated with high protein intake in individuals with grade III obesity in the Brazilian population [94].

Among the loci most consistently associated with obesity is the *FTO* (Fat Mass and Obesity-associated) gene, in which variants such as rs9939609 (T > A) are related to higher BMI, greater predisposition to weight gain, and alterations in IR markers [83,95]. At a mechanistic level, experimental evidence derived from skeletal muscle cell models indicates that the A allele, considered a risk factor, can modulate pathways involved in energy metabolism and insulin signaling in pro-inflammatory contexts [96]. Furthermore, a population-based study with the Mexican population revealed that individuals with the TT genotype of rs9939609 were more likely to exhibit metabolic alterations, including in the homeostatic model assessment of insulin resistance (HOMA-IR) index and hypertriglyceridemia, and a 2.5 times greater odds ratio of hyperlipidemia compared to carriers of the AA + AT genotypes [95].

The rs1801282 polymorphism (risk allele G) in *PPARG* (peroxisome proliferator-activated receptor gamma), a gene central to adipogenesis and insulin sensitivity, has also been associated with metabolic alterations and risk of prediabetes in different populations due to dysfunctional activity of the PPARγ2 receptor [97].

In addition to the genes classically implicated in insulin signaling, variants in *IRS1* (rs1801278, rs2943641, and rs7578326) have been reported in reviews that synthesize loci associated with IR and components of MetS, reinforcing their role as modulators of glucose and lipid metabolism at the population level [29].

In the inflammatory axis, evidence shows that the C alleles of the rs1800795 and rs1800796 polymorphisms in the *IL6* gene are associated with both the risk of obesity and phenotypes of low-grade chronic inflammation, with implications for metabolic homeostasis and insulin sensitivity, as demonstrated in recent meta-analyses [98,99]. In this context, the SNPs rs1800629 (risk allele A) and rs361525 (risk allele A) in the *TNFA* gene have been linked to the modulation of TNF-α expression and markers of IR, highlighting possible interactions with dietary patterns and diet composition [22,29,100].

In a meta-analysis, the results of the studies demonstrated a strong correlation in the Caucasian and Asian population between the G allele of the rs738409 SNP of the patatin-like phospholipase domain-containing protein 3 (*PNPLA3*) gene, which encodes a protein (a triacylglycerol lipase) that mediates triacylglycerol hydrolysis in adipocytes, and the risk of liver cirrhosis, in which chronic inflammation is involved in the progression of this disease through the activation of inflammatory signaling pathways mediated by ROS, resulting in NF-κB activation via IKK/IκB in hepatocytes [101,102].

Additionally, variants in the *TLR4* gene, such as the rs4986790, rs4986791, rs10983755, and rs5030717 polymorphisms, show relevant biological plausibility by influencing the activation of TLR4- and NF-κB-dependent inflammatory pathways, establishing a connection between metabolic endotoxemia and IR. Observational studies have described associations of these variants with T2D and metabolic inflammatory phenotypes, suggesting their involvement at the interface between inflammation, metabolism, and cardiometabolic risk [103,104]. A broader overview of additional SNPs associated with these pathways is summarized in Supplementary Table S1.

In this context, IR is a complex phenotype resulting from the interaction of genetic predisposition, nutritional environment, and low-grade chronic inflammation. Identifying SNPs associated with adiposity, insulin signaling, and the inflammatory response has contributed to elucidating the biological mechanisms that drive individual variations in cardiometabolic risk. These findings reinforce the polygenic nature of IR and underscore the potential for personalized nutritional approaches and risk stratification based on genetic profiles.

Because most cardiometabolic and metabolic traits have a complex and polygenic architecture, the interpretation of isolated SNP associations should be made with caution. Individual variants identified through GWAS generally have small effect sizes and, when analyzed separately, may provide limited and sometimes inconsistent information for disease risk prediction. In this context, genetic risk scores and polygenic risk scores have emerged as more integrative approaches because they aggregate the cumulative contribution of multiple risk alleles into a single quantitative measure of genetic susceptibility. Typically, these scores are calculated as a weighted sum of allele counts, with weights derived from effect-size estimates obtained in GWAS or association studies. Therefore, rather than replacing biological interpretation of individual variants, genetic scores provide a complementary framework that may better reflect the cumulative and polygenic nature of obesity, type 2 diabetes, dyslipidemia, MASLD, and other cardiometabolic phenotypes [105,106,107].

Nevertheless, the use of genetic or polygenic scores also requires a critical interpretation. Their predictive performance depends on the variants selected, the effect-size estimates used as weights, linkage disequilibrium structure, sample size, population ancestry, phenotype definition, and external validation. Current evidence also indicates important limitations, including reduced transferability across ancestries, challenges in admixed populations, heterogeneity in polygenic risk score construction and reporting, and incomplete incorporation of environmental exposures, lifestyle factors, and gene–environment or gene–diet interactions. Thus, although genetic scores may reduce some limitations of single-SNP analyses by capturing cumulative genetic susceptibility, they should not be interpreted as deterministic predictors or as substitutes for clinical, biochemical, anthropometric, inflammatory, and lifestyle-related risk factors. Future studies should prioritize transparent reporting, population-specific or multi-ancestry validation, and integrated models combining genetic susceptibility with environmental and metabolic data [105,106,107,108].

Another methodological issue that should be considered when interpreting SNP-based associations is the winner’s curse, a selection-related bias in which genetic variants identified as statistically significant in discovery studies tend to have overestimated effect sizes compared with their true effects or subsequent replication estimates. This bias is particularly relevant in studies with limited sample size, low statistical power, multiple testing, or lack of independent replication and may contribute to apparently inconsistent findings across populations and study designs. Therefore, associations involving isolated polymorphisms should be interpreted cautiously and, when possible, supported by adequately powered replication studies, ancestry-aware analyses, corrections for multiple comparisons, and integrated approaches such as genetic or polygenic risk scores [109,110].

Accordingly, research on polymorphisms related to obesity and cardiometabolic disorders should prioritize large-scale, longitudinal, and ancestrally diverse studies capable of evaluating common and rare variants, as well as their interactions with modifiable exposures such as diet and lifestyle. Recent genome-wide gene–diet interaction analyses have shown that reproducible interactions between macronutrient intake and glycemic traits remain difficult to detect, partly due to dietary measurement error, limited statistical power, population heterogeneity, and an insufficient assessment of rare genetic variation [111]. Therefore, studies should improve dietary exposure assessment through repeated measures, validated instruments, and, when possible, objective nutritional biomarkers, while also incorporating whole-genome sequencing and adequately powered samples to investigate gene–diet interactions across the allele-frequency spectrum [111]. In parallel, recent multi-ancestry polygenic prediction studies for body mass index and obesity suggest that polygenic scores may improve early risk prediction and help identify individuals with greater genetic susceptibility across the life course; however, their predictive performance still varies substantially across ancestral groups [112]. Thus, future research should focus on multi-ancestry validation, population-specific calibration, the integration of genetic scores with clinical, metabolic, dietary, and lifestyle-related risk factors, and evaluation within longitudinal cohorts and dietary intervention trials [112]. These approaches may help clarify inconsistent findings from isolated SNP analyses and support the responsible translation of genetic information into precision nutrition strategies.

### 3.3. MicroRNAs Related to Insulin Resistance, Inflammation, and Obesity

In recent decades, advances in proteomics and genomics have broadened our understanding of the molecular mechanisms underlying IR, demonstrating that, alongside genetic determinants, epigenetic mechanisms play a central role in modulating insulin signaling and the inflammatory response associated with obesity [13,27]. Among these mechanisms, miRNAs stand out as post-transcriptional regulators capable of integrating nutritional, metabolic, lipotoxic, and inflammatory stimuli, thereby modulating gene networks involved in energy homeostasis [13,27,28].

**Figure 3:**
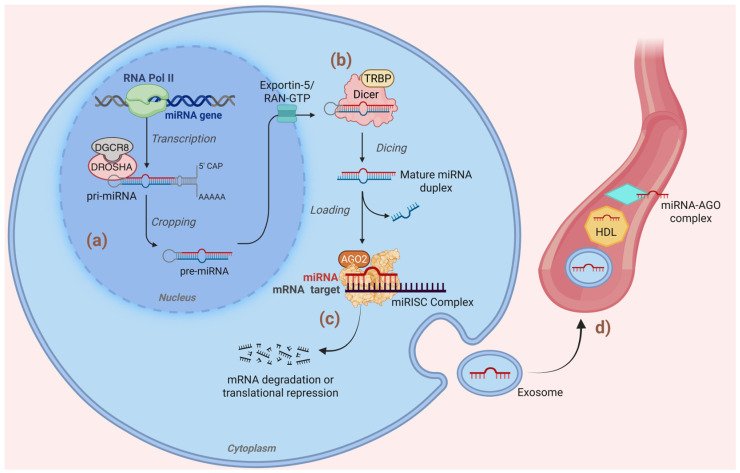
Canonical pathway of microRNA biogenesis, processing, and cellular release mechanisms. (**a**) Nuclear processing: miRNA genes are transcribed by RNA polymerase II, resulting in a primary transcript (pri-miRNA). In the nucleus, this transcript is processed by the microprocessor complex, composed of ribonuclease III (DROSHA) and the DiGeorge syndrome critical region 8 (DGCR8) protein, generating the miRNA precursor (pre-miRNA). (**b**) Cytoplasmic export and maturation: The pre-miRNA is exported to the cytoplasm by the Exportin-5/Ran-GTP complex. In the cytoplasm, it is cleaved by endonuclease RNase III (DICER), in association with the Tar RNA-binding protein (TRBP), resulting in an miRNA duplex. (**c**) Gene silencing: One of the strands of the duplex (guide strand) is incorporated into the RNA-induced silencing complex (miRISC), which contains the argonaute 2 (AGO2) protein. This complex recognizes partially complementary sequences in the target messenger RNA (mRNA), promoting its degradation and/or translational repression. (**d**) Extracellular secretion pathways: After maturation, miRNAs can be exported to the extracellular environment and systemic circulation. This process occurs through encapsulation in extracellular vesicles, such as endosomal exosomes, and association with RNA-binding proteins, such as AGO2, or high-density lipoprotein (HDL). Source: Adapted from Quintanilha et al. [16], with permission. Created by the authors using BioRender. Corsi, G. (2026) https://BioRender.com/jkrzzog.

MiRNAs are small non-coding ribonucleic acid molecules, approximately 22 nucleotides in length, that regulate gene expression post-transcriptionally by binding to the 3′ untranslated regions or 5′ untranslated regions of messenger RNA (mRNA), promoting transcript cleavage, translational repression, or degradation mediated by deadenylation and decapping [113,114]. In mammals, the canonical miRNA biogenesis pathway begins in the nucleus with the transcription of a primary miRNA by the enzyme RNA polymerase II, followed by nuclear processing by the RNase III enzyme complex (DROSHA) and its cofactor DiGeorge 8 (DGCR8), generating a precursor miRNA. The miRNA precursor is then exported to the cytoplasm by the exportin-5 protein with the aid of the nuclear transport protein (RAN-GTP). In the cytoplasm, it is cleaved by the endonuclease RNase III (DICER), in association with the TAR RNA-binding protein (TRBP), resulting in an miRNA duplex. This duplex is incorporated into the RNA-induced silencing complex (RISC), where argonaute family proteins promote the removal of the complementary strand, resulting in the functional mature miRNA (Figure 3) [16,113,114].

In addition to their intracellular action, miRNAs may be secreted associated with ribonucleoprotein complexes linked to the argonaute proteins, carried by lipoproteins, associated with nucleophosmin 1, or encapsulated in extracellular vesicles, such as exosomes and microvesicles [16,113,115]. When detectable in biological fluids, they are referred to as circulating miRNAs, expanding their regulatory role to the systemic level, enabling interorgan communication and modulation of insulin sensitivity in tissues distant from their site of origin (Figure 3) [113,114].

In this context, increased serum concentrations of miR-122, miR-29a, and miR-128-1 have been reported to be associated with elevated HOMA-IR, dyslipidemia, and alterations in basal energy metabolism [116,117,118,119].

Experimental evidence in murine models indicates that miR-122 acts as a mediator of hepatic IR by targeting the insulin-like growth factor 1 receptor (IGF-1R) in hepatocytes, thereby attenuating PI3K/AKT signaling and promoting gluconeogenesis [120]. Furthermore, in silico analyses have identified *PRKAB1*, a regulatory subunit of AMPK, as a predicted target of miR-122 [121]. Moreover, other findings suggest that this miRNA may impair AMPK activity by repressing sirtuin 1, which compromises the activation of liver kinase B1 (*LKB1*) and, consequently, AMPK itself [122]. Given the role of AMPK in inhibiting lipogenesis and stimulating fatty acid oxidation, its repression may contribute to the exacerbation of IR [122,123].

Moreover, miR-29a may be secreted by adipose tissue macrophages and transferred via exosomes to myocytes and hepatocytes, where it represses peroxisome proliferator-activated receptor-delta (*PPARD*), impairing the expression of genes involved in fatty acid oxidation in adipose tissue and skeletal muscle. Attenuation of this pathway favors dyslipidemias and impaired insulin sensitivity [124,125].

In turn, miR-128-1 acts as a regulator of energy balance: while its overexpression is related to an energy-conserving phenotype in humans, its ablation or inhibition by anti-miR oligonucleotides in murine models of obesity resulted in a significant increase in energy expenditure, reduction in adiposity, and improvement in insulin sensitivity [119,126]. These effects are mediated by derepression of miR-128-1 target genes, including PR domain containing 16 (*PRDM16*), peroxisome proliferator-activated receptor gamma coactivator 1 alpha (*PPARGC1A*) and peroxisome proliferator-activated receptor alpha (*PPARA*), which are regulators of mitochondrial biogenesis and thermogenesis in metabolically active tissues [126].

Dysfunctional WAT itself acts as an active source of miRNAs that amplify metabolic inflammation and contribute to IR [27,127,128,129]. Hypertrophic adipocytes secrete exosomal miRNAs, such as miR-34a, which, when internalized by resident macrophages, repress the transcription factor Krüppel-like 4 (KLF4), impairing anti-inflammatory M2 polarization and favoring the pro-inflammatory M1 phenotype, thereby contributing to the induction of pro-inflammatory cytokines and local fibrosis [130]. Similarly, the overexpression of miR-802 in adipocytes promotes the activation of canonical NF-κB pathways by repressing its negative regulator TRAF3, indirectly increasing the expression of chemokines and SREBP1, and intensifying M1 macrophage polarization [129].

Conversely, miRNAs such as miR-126 and miR-146a exert a negative regulatory function on metabolic inflammation by silencing *IRAK1* and *TRAF6*, thereby attenuating NF-κB activation [131,132,133,134,135]. The reduced circulating levels of these miRNAs in individuals with T2D and prediabetes indicate their relevance as potential biomarkers of metabolic dysfunction [131,132,133,136]. However, the regulation of miR-146a appears to be dependent on the biological context. The study by Lopez et al. [137] demonstrated that, in abdominal adipose tissue, miR-146a expression is significantly elevated in individuals with obesity and diabetes and is positively correlated with body fat percentage and with the antiangiogenic factor secreted frizzled-related protein 4 (SFRP4) [137]. In this regard, the authors suggested the existence of a negative feedback mechanism in which increased miRNA expression may represent an attempt to counterbalance the inflammation and capillary rarefaction associated with the dysfunctional expansion of abdominal fat [137].

The IR imposes greater functional demand on pancreatic β cells, whose compensatory capacity is essential for maintaining glycemic homeostasis [138]. In this scenario, miR-375, abundantly expressed in these cells, regulates the expression of genes involved in maintaining β-cell mass and function [139,140]. Under conditions of metabolic and inflammatory stress, its overexpression compromises PDK1/AKT signaling and reduces activity of the mechanistic target of rapamycin complex 2 (*mTORC2*) through repression of MAPK associated protein 1 (MAPKAP1), negatively impacting cell proliferation and survival [140,141].

Consistent with this functional relevance, elevated serum and plasma levels of miR-375 have been associated with T2D and its vascular complications in cross-sectional studies, suggesting its potential as a non-invasive biomarker [142,143]. However, in a clinical trial of a dietary intervention for weight loss, increased serum levels of miR-375-3p were associated with greater reductions in visceral fat, distal subcutaneous fat, and intrahepatic fat content, compartments closely related to IR [144]. Thus, these divergent findings suggest that the biological significance of miR-375 depends on the metabolic context.

Taken together, these findings support the notion that miRNAs constitute a sophisticated regulatory layer that integrates metabolic and inflammatory signals across diverse tissues. This integration increases the complexity of the pathophysiology underlying obesity associated with IR and metabolic inflammation. Nevertheless, although mechanistic evidence supports their functional relevance, the heterogeneity observed among clinical and experimental studies—compounded by tissue specificity, disease progression, and inflammatory profiles—warrants caution in the translational interpretation of these findings.

### 3.4. DNA Methylation Related to Insulin Resistance, Inflammation, and Obesity

DNA methylation is an epigenetic mechanism of gene regulation that has been associated with the onset and progression of metabolic diseases, modulating pathways related to IR, inflammation, and adiposity [78,145,146]. This mechanism is characterized by the covalent binding of a methyl group to specific regions of DNA [145]. It occurs at the carbon-5 position of cytosine residues adjacent to a guanine nucleotide, forming 5-methylcytosine within CpG dinucleotides. CpG-dense regions, known as CpG islands, are frequently located within promoter regions and, under physiological conditions, are predominantly hypomethylated. Aberrant methylation of these regions may inhibit the binding of transcription factors, ultimately leading to repression of gene expression [145,146]. This process is mediated by the coordinated action of enzymatic machinery. DNA methyltransferases catalyze the addition of methyl groups, whereas a group of proteins known as ten-eleven translocation (TET) enzymes act as demethylases by removing them, highlighting the dynamic and plastic nature of this regulatory mechanism [78,147].

Under homeostatic conditions, specific genomic regions exhibit either hyper- or hypomethylation, reflecting the balanced activity of these enzymes [78]. Regarding DNA methylation, diet plays a pivotal role, as the universal methyl donor for DNA methyltransferases is S-adenosylmethionine (SAM), whose synthesis depends on the dietary intake of the precursor amino acid methionine and B-complex vitamins—particularly folate—that act as cofactors in this reaction. This dependence highlights the relevance of dietary patterns in the direct regulation of this epigenetic machinery [148]. Disruption of this balance, driven, for example, by suboptimal dietary patterns, may promote the development of metabolic diseases [147]. Concomitantly, pathophysiological alterations of pathways related to obesity and chronic inflammation may also induce changes in DNA methylation patterns [145].

In this context, Nadiger et al. [149] conducted a systematic review focused on mapping DNA methylation associated with T2D and identified 130 differentially methylated loci in individuals with T2D, particularly in tissues crucial for metabolic homeostasis, including adipose tissue, liver, pancreas, skeletal muscle, and blood. The genes *ABCG1*, *TXNIP*, *PTPRN2*, and *PPARGC1A* consistently exhibited distinct methylation patterns across multiple studies, with *TXNIP* being the most frequently reported gene. Notably, hypomethylation of *TXNIP* in T2D patients was consistent across all reviewed studies. Under hypoxic conditions arising from metabolic dysfunction, *TXNIP* expression is upregulated, where it functions as a critical glucose sensor across multiple tissues. In pancreatic β-cells, glucose-derived metabolites, especially glucose and fructose, promote activation of ChREBP, which binds to the *TXNIP* promoter region, increasing its transcription. This process is associated with the induction of β-cell apoptosis in response to hyperglycemia. Furthermore, increased *TXNIP* expression in skeletal muscle, adipose tissue, and liver is associated with impaired glucose uptake [150]. In this context, hypomethylation of *TXNIP* in T2D, with consequent attenuation of epigenetic repression, is consistent with mechanisms that may sustain the underlying pathophysiological state.

Another equally relevant mechanism in the pathogenesis of metabolic diseases is lipid metabolism dysfunction [151]. Alterations in DNA methylation patterns in SAT have been associated with circulating triglyceride concentrations [152], suggesting a potential role for epigenetic regulation in modulating the lipid profile. In this context, adipokines, which are key regulators of energy metabolism whose circulating levels reflect adipose tissue dysfunction, also integrate into this pathophysiological axis. Sinke et al. [151] conducted a large-scale epigenome-wide meta-analysis and identified 73 CpG sites associated with adiponectin and 211 CpG sites related to leptin. Among these, loci involved in lipid transport (*ABCG1*), fatty acid oxidation (*CPT1A*), and cholesterol biosynthesis (*DHCR24*) were highlighted, underscoring the role of DNA methylation in regulating adipokine-mediated central pathways of lipid metabolism [151].

Moreover, two CpG sites were identified whose methylation appears to act upstream in the pathogenesis of T2D: one located in the *ADIPOQ* gene, which encodes adiponectin, and another associated with *SREBF1* expression, a key regulator of lipid homeostasis [151]. Additionally, DNA methylation in specific genes within SAT has been proposed as a potential partial mediator of the effect of circulating lipids on peripheral IR, reinforcing the potential role of epigenetic regulation as a mechanistic link in the development of cardiometabolic diseases [152].

Another inherent feature of DNA methylation is its tissue-specific nature. In a pathophysiological context, the same gene may be either hypo- or hypermethylated depending on the tissue evaluated [149,152]. Beyond this gene-specific variation, global methylation patterns differ across tissues, reflecting distinct regulatory programs and biological pathways. In WAT, for example, aberrant DNA methylation changes on SAT have been associated with pathways involved in glycolipid metabolism and IR, whereas in VAT such alterations have been linked to processes of cell death, apoptosis, and cell cycle regulation [153]. These alterations in WAT are particularly relevant in the context of obesity, in which hypertrophic and hyperplastic adipocytes promote a hypoxic environment that culminates in the activation of pro-inflammatory pathways [145].

Adipocytes from obese women showed downregulation of the *SLC19A1* gene, which is responsible for folate transport, resulting in DNA hypermethylation and altered expression of inflammatory genes, particularly *CCL2*, suggesting a role for adipocyte DNA methylation in metabolic disease development while also highlighting the relevance of folate in this regulatory process [154]. Additionally, alterations in DNA methylation patterns of genes involved in energy metabolism and inflammation in VAT have been associated with the etiology of MetS [146]. Aberrant DNA methylation in inflammatory genes—such as *SOCS1* in blood cells, *CCL20* on SAT, *JUN* in VAT, and *PLA2G2A* in liver tissue—has been identified in patients with obesity and T2D [153]. Furthermore, hypomethylation of *TNFA* observed in individuals with MetS may reflect WAT dysfunction, considering that the expression of this cytokine is largely dependent on macrophages, which, under conditions of sustained chronic inflammation, become functionally dysregulated [146].

However, it is important to note that the studies still exhibit considerable methodological heterogeneity. For example, while some investigations assessed individuals not receiving medication [152], others included participants undergoing pharmacological treatment [146], which may influence the observed methylation profiles. Furthermore, the available evidence is derived predominantly from European populations [146,149,151,152], limiting the generalizability of the findings to other ethnic groups. Additional limitations include the relatively small sample sizes of some studies [146,152,153,154] and the predominantly cross-sectional design adopted in part of the literature [146,153], both of which may reduce the precision of the estimates and hinder the assessment of temporal relationships, respectively. Consequently, it remains controversial whether DNA methylation plays a causal role in metabolic dysfunction or primarily reflects the metabolic state associated with the disease. Moreover, the scarcity of functional validation analyses warrants caution when interpreting these findings, as alterations in DNA methylation do not necessarily translate into changes in gene expression [149]. Therefore, although the available evidence supports a potential role for DNA methylation in the pathophysiology of metabolic diseases, prospective studies involving more diverse populations and rigorous functional validation are needed to determine whether DNA methylation changes preceded by disease onset or result from disease progression, thereby clarifying their biological relevance and facilitating their clinical translation.

## 4. Nutritional Strategies in the Context of Nutritional Genomics

### 4.1. Nutrigenomics

Nutrigenomics investigates how diet, dietary components or dietary patterns affect gene expression at the molecular, pre-transcriptional, post-transcriptional, and translational levels and, consequently, health [19,23]. This knowledge may contribute to personalized nutritional strategies, as well as disease prevention and treatment [23,155,156].

Omega-3 polyunsaturated fatty acids (PUFAs), specifically alpha-linolenic acid (ALA), eicosapentaenoic acid (EPA), and docosahexaenoic acid (DHA), are prominent bioactive lipids recognized for mitigating cardiometabolic risk through complex molecular mechanisms [157,158]. These nutrients modulate inflammatory markers, oxidative stress, and insulin sensitivity by suppressing the expression and secretion of pro-inflammatory cytokines, such as IL-1β, IL-6, and TNF-α, as well as ICAM-1 and VCAM-1 [158,159,160,161].

Omega-3 fatty acids, particularly EPA and DHA, bind to the cell surface receptor, free fatty acid receptors (FFAR) 4, also known as GPR120, a G protein-coupled receptor [161,162,163,164]. This interaction promotes the recruitment of β-arrestin 2, leading to the formation of the GPR120/β-arrestin 2 complex, which associates with the adaptor protein TAB1. The sequestration of TAB1 prevents its interaction with TAK1, resulting in the inhibition of intracellular signaling pathways IKK/MAPK and JNK. Consequently, the activation of transcription factors NF-κB and AP-1, which are associated with the inflammatory response, is reduced [161,162,163,165]. FFAR4/GPR120 also inhibits activation of the NLRP3 inflammasome, suppressing caspase-1 activation and reducing the subsequent secretion of inflammatory cytokines IL-1β and IL-18 [161,162,163,165,166].

Additionally, a meta-analysis evaluating ten studies on the effects of omega-3 fatty acid supplementation on *PPARG* gene expression demonstrated that this intervention significantly increases the expression of this gene compared to the control group, promoting the production of anti-inflammatory adipokines, such as adiponectin, and suppressing NF-κB activation [163,167]. However, it is important to note that a high level of heterogeneity was observed among the studies included in the meta-analysis, including differences in participants’ health conditions, BMI ranges, omega-3 fatty acid dosages, and intervention durations. Therefore, the findings should be interpreted with caution, as these factors may have influenced the overall results of the meta-analysis [167].

PPARs are nuclear receptors that regulate genes involved in lipid and glucose metabolism, energy balance, and inflammation, with PPARγ being one of the most relevant. PPARγ, once activated, binds to specific DNA sequences and regulates adipocyte differentiation, lipid storage, and adipokine secretion. Alterations in PPARγ expression and function contribute to IR and obesity [159,167,168]. Omega-3 fatty acids also act as a nuclear suppressor of SREBP-1, improving lipid profile and modulating inflammation [159,160,169].

Among dietary components, BCs exert biological activity in the body. Found mainly in fruits, vegetables, spices, nuts, and grains, plant-derived BCs are referred to as phytochemicals. These compounds are present in foods as part of plants’ defense mechanisms against environmental stressors. In the human body, they provide health benefits due to their antimicrobial, antiviral, anti-inflammatory, antioxidant, and immunomodulatory properties [170,171,172,173].

There are more than one thousand phytochemicals, which are classified into subgroups. Polyphenols represent a major category of BCs with phenolic structures and include main subclasses such as flavonoids, stilbenes, phenolic acids, and lignans [170,171,172]. BCs can act through different mechanisms and molecular targets, thereby modulating the inflammatory response, reducing biomarkers, and providing health benefits [24,173,174,175].

Curcumin is a yellow phenolic pigment extracted from the rhizomes of *Curcuma longa* L., a member of the curcuminoid family. This BC has been used in the management of various conditions, including respiratory diseases, inflammation, liver disorders, diabetes, rheumatism, and certain types of cancer, due to its multiple biological properties. Curcumin exhibits antioxidant, antidiabetic, anticancer, anti-inflammatory, antibacterial, antiviral, and antifungal effects [24,173,175,176,177]. A meta-analysis of clinical trials demonstrated a protective effect of curcumin against hyperglycemia and inflammation in T2D, reducing fasting glucose, glycated hemoglobin, and C-reactive protein levels [177].

Curcumin modulates inflammatory signaling pathways of NF-κB and AP-1, inhibiting the production of inflammatory mediators. It can downregulate the expression of TLR4 and MAPK [174,178,179]. Additionally, curcumin has been shown to upregulate and activate AMPK, which negatively regulates NF-κB [177,179]. Another mechanism involves the inhibition of IKK activity and prevention of phosphorylation and degradation of IκB-α [177,179,180,181]. Furthermore, curcumin inhibits the NLRP3 inflammasome, reducing IL-1β and IL-18 levels [175,178,182].

Catechin is a polyphenol belonging to the flavonoid subgroup, naturally present in foods such as fruits (grapes, apples, strawberries), chocolate, and especially teas, particularly green tea [24,174,183]. Catechins are a group of polyphenolic compounds that include catechin, epicatechin, gallocatechin, epigallocatechin, catechin gallate, epicatechin gallate, gallocatechin gallate and epigallocatequin gallate, the latter being the most abundant and widely studied [183,184]. Their beneficial properties include immunomodulatory, anti-inflammatory, antioxidant, antihypercholesterolemic, antimicrobial, and anticancer effects [24,183,184,185].

Due to their chemical structure, catechins act as free radical scavengers and inhibit the formation of ROS [183,184]. Epigallocatequin gallate exerts anti-inflammatory effects because it suppresses the expression of TLR4 and the activation of NF-κB, inhibiting the phosphorylation of IκB [174,183,184,185]. Another mechanism of catechin involves inhibition of MAPK activity, particularly p38 phosphorylation, thereby suppressing NF-κB-mediated inflammatory pathways [24,176,180,183,184,185].

Resveratrol is a polyphenol belonging to the stilbene subgroup, naturally found in foods such as grapes, wine, berries, and peanuts [24,174,186]. Structurally, it consists of two aromatic rings linked by a methylene bridge and exists in two isoforms: trans-resveratrol (more stable) and cis-resveratrol [24,186]. Resveratrol exhibits strong anti-inflammatory and antioxidant effects [186,187].

Resveratrol exerts anti-inflammatory effects through multiple mechanisms, including downregulation of TLR4 expression [174,175] inhibition of NF-κB activation via reduced degradation of its inhibitory protein, IκB-α [175,181]. Its effects are also attributed to the activation of AMPK and sirtuin 1, both of which act as important inhibitors of the NLRP3 inflammasome and NF-κB [173,174,182,186,188,189].

Adherence to healthy dietary habits is strongly correlated with a diminished risk of chronic diseases. The Mediterranean Diet (MedDiet) stands out as a prominent dietary pattern capable of modulating key molecular mechanisms and exerting robust nutrigenomic effects. By influencing systemic pathways related to inflammation, oxidative stress, and lipid metabolism, the MedDiet serves as a potent epigenetic and transcriptional regulator. Substantial scientific evidence supports its therapeutic benefits and its recognition as a primary nutritional intervention for mitigating the incidence of CVD, T2D, MetS, obesity, and cancer [190,191,192,193,194,195,196,197,198].

The MedDiet refers to the traditional dietary pattern of populations living along the Mediterranean coast. It is characterized by high consumption of olive oil, fruits, vegetables, whole grains, legumes, nuts, and low-fat dairy products; moderate alcohol intake, mainly red wine with meals; and low consumption of red meat [190,191,195]. This composition provides essential and beneficial components such as omega-3 fatty acids, monounsaturated fats, BCs (including polyphenols), fiber, vitamins, and minerals [191,199].

Overall, due to the combined effects of these components, this dietary pattern is associated with reduced oxidative stress, inflammation, thrombosis, and endothelial dysfunction, as well as improved insulin sensitivity, lipid profiles, and gut microbiota [191,198]. BCs present in the MedDiet contribute to the inhibition of cyclooxygenase enzymes (COX-1 and COX-2) and, together with omega-3, reduce ROS levels and downregulate NF-κB signaling, resulting in decreased expression of pro-inflammatory factors such as IL-1β, IL-6, and TNF-α [190,191,192].

This dietary pattern is also associated with beneficial effects on gut microbiota, modulating its composition and diversity. It promotes an increase in beneficial bacteria such as *Bifidobacterium* and a reduction in potentially harmful bacteria such as *Firmicutes*. This modulation enhances the production of metabolites such as short-chain fatty acids (acetate, propionate, and butyrate), which exert immunomodulatory and anti-inflammatory effects and contribute to overall health [191,198,200].

Short-chain fatty acids exert their beneficial effects mainly through the activation of G-protein coupled receptors (GPCRs), particularly GPR41 (free fatty acid receptor 3), GPR43 (free fatty acid receptor 2) and GPR109A [201,202]. These receptors are expressed in the white adipose tissue, the liver, skeletal muscles, the gastrointestinal tract and immune cells, including macrophages [201]. Activation of GPR43 and GPR109A promotes potassium (K^+^) efflux and membrane hyperpolarization, leading to the activation of the NLRP3 inflammasome and the conversion of pro-IL-18 into their active form [203,204]. IL-18 is generally classified as a pro-inflammatory cytokine; it plays a key role in maintaining intestinal epithelial integrity and promoting epithelial repair under physiological conditions. However, it is important to note that this mechanism has been demonstrated primarily in mice models [204]. Animal and in vitro studies have also shown that short-chain fatty acids, through the activation of GPCR signaling pathways, inhibit NF-κB activation, thereby reducing the production of pro-inflammatory cytokines, including TNF-α and IL-6. Proposed mechanisms to this effect include increased IκB expression, which prevents NF-κB activation, and reduced NF-κB nuclear translocation, thereby limiting the transcription of inflammatory genes [202,205,206].

Overall, studies investigating how dietary components or dietary patterns affect gene expression exhibit substantial methodological heterogeneity, including differences in study design, participant characteristics, dietary interventions, dosages, intervention duration, and the bioavailability of the compounds. In addition, genetic variability among individuals may influence the results. Therefore, the studies should be interpreted with caution, as these factors may limit the generalizability of the results. 

### 4.2. Nutrigenetics

Recent advances in nutrigenetics and precision nutrition have strengthened the understanding that interindividual variability in metabolic responses to diet is largely influenced by genetic background, supporting the development of individualized strategies for cardiometabolic disease prevention and management [18]. In this context, multi-omics approaches integrating genomics, metabolomics, and microbiome data further highlight that individuals respond differently to the same dietary interventions, reinforcing the need for personalized nutrition [207].

The ASPIRE-DNA study, a pilot randomized controlled trial, investigated the effects of DNA-based personalized nutrition in individuals with non-diabetic hyperglycemia over 26 weeks. While no significant short-term changes were observed, longer-term results demonstrated improvements in glycemic control, including reductions in fasting plasma glucose and HbA1c in participants receiving genotype-based dietary advice compared to standard care. These interventions were personalized according to genetic variants related to macronutrient metabolism, supporting the concept that personalized dietary strategies may enhance metabolic outcomes. However, findings should be interpreted with caution due to the relatively small sample size [208].

Similarly, personalized nutrition based on dietary intake, phenotype, and genotype led to significant improvements in dietary behaviors such as reductions in the consumption of red meat (−5.48 g/day), salt (−0.65 g/day), and saturated fat (−1.14% of energy), alongside increased folate intake (+29.6 µg/day) and improved overall diet quality. However, the inclusion of phenotypic and genotypic data did not provide additional benefits beyond personalization based on an assessment of the individual’s current diet [209].

From a mechanistic perspective, genetic variants can alter metabolic fluxes and intracellular signaling cascades, influencing the phenotype of a SNP that can vary according to the ancestry of the population. A study carried out in a Mexican population demonstrated that individuals carrying the homozygous TT genotype of SNP rs9939609 of the *FTO* gene showed a greater likelihood of developing hyperglycemia, hypertriglyceridemia and IR. Although no significant differences in food intake were reported between genotypes, individuals carrying the TT genotype showed greater susceptibility to metabolic dysfunction, regardless of BMI, suggesting that this genetic variant may present increased vulnerability when exposed to obesogenic environments [95].

Metabolic traits and the risk of cardiometabolic diseases vary among populations, largely influenced by SNPs in the *IRS1* gene, which plays a critical role in glucose homeostasis [20,210,211]. Phenotypic expression often depends on dietary components and physiological status. For instance, carriers of the G allele of SNP rs7578326 exhibit a lower risk of MetS than non-carriers when monounsaturated fatty acid intake is below average. Similarly, carriers of the T allele of SNP rs2943641 have a significantly lower risk of MetS at a low dietary saturated fatty acid-to-carbohydrate ratio [211]. These findings corroborate the locus’s role in insulin signaling and susceptibility to endocrine dysfunctions. Conversely, individuals carrying the CT and CC genotypes of SNP rs2943641 are associated with elevated fasting insulin concentrations [20].

Genes related to metabolic regulation, such as *PPARG* (rs1801282) and *TCF7L2* (rs7903146), have shown significant genetic associations with the development of early-stage metabolic disorders. The *PPARG* gene plays a role in regulating glucose and lipid homeostasis, as well as the differentiation, maturation, and proper functioning of adipocytes. Individuals of Kazakh ethnicity carrying the *PPARG* GG genotype exhibited an increased risk of developing pre-diabetes, and this association is heightened when combined with factors such as variations in birth weight and a family history of cardiometabolic disease [97].

Individuals homozygous for the risk allele (G) of SNP rs738409 in the *PNPLA3* gene were 105% more likely to develop MASLD compared to those with other genotypes [212]. However, IR is an integral and necessary component for the manifestation of MASLD in both carriers and non-carriers of the G risk allele (rs738409) in the *PNPLA3* gene [213]. These results corroborate the role of SNPs as a susceptibility factor that exacerbates liver damage rather than acting as a standalone cause, contrasting with systemic metabolic conditions like obesity and diabetes.

Variants in genes such as *TCF7L2*—one of the most robust genetic determinants associated with T2D risk—highlight the clinical importance of molecular genetics in the regulation of glucose homeostasis [85,86,87,89]. These SNPs impair insulin secretion and incretin signaling [86,87]; however, emerging evidence suggests that dietary patterns can modulate their phenotypic expression, reinforcing the need for genotype-guided nutritional strategies [89]. A crossover clinical study involving overweight or obese participants evaluated the impact of the MedDiet versus a low-fat diet over one-week intervals. A significant interaction was identified between the rs7903146 polymorphism in the *TCF7L2* gene and dietary patterns; the CC genotype—associated with lower susceptibility to T2D—demonstrated an efficient metabolic response to the Mediterranean diet, resulting in a combined reduction in plasma SFA and monounsaturated fatty acid levels [214]. Conversely, individuals carrying the high-risk TT genotype showed greater vulnerability to disrupted lipid homeostasis and increased levels of atherogenic VLDL particles specifically during the low-fat diet. This challenges the necessity of severe lipid restriction for this group, as adopting the MedDiet may counteract this genetic risk and protect the cardiovascular health of T-allele carriers [215]. Therefore, these findings should not be interpreted as evidence that the TT genotype benefits from a high-fat diet per se. Rather, they suggest that a Mediterranean dietary pattern—characterized by higher unsaturated fat content, lower carbohydrate proportion, and slightly greater fiber intake—may attenuate the adverse triglyceride-rich lipoprotein response observed among TT carriers under low-fat/high-carbohydrate conditions. This interpretation reconciles the apparently paradoxical finding with the established high-risk profile of the TT genotype, while also emphasizing that the study evaluated short-term lipid remodeling, not GLP-1 secretion or T2D incidence. Moreover, because the formal genotype-by-diet interaction was not statistically significant, these findings should be considered hypothesis-generating and interpreted with caution.

In a randomized, double-blind, parallel-group clinical trial aimed at determining the impact of a fiber supplement (glucomannan, inulin, and psyllium) or a placebo over 180 days on the weight and body composition of obese individuals carrying at least one minority allele in weight-control-related polymorphisms—*FTO* (rs9939609; T > A), *LEP* (rs2167270; G > A), *LEPR* (rs1137101; A > G; Gln223Arg), and *MC4R* (rs17782313; T > C)—weight loss and appetite changes were observed after supplementation in this population. Although all genetic groups benefited from the fiber intervention, homozygous carriers of minority alleles showed greater reductions in body weight (−3.2%; 95% CI: −4.9% to −1.6%; *p* < 0.01) and BMI (−1.2 kg/m^2^; 95% CI: −2.0 to −0.4; *p* < 0.01) compared to heterozygous carriers of the allele [216].

Furthermore, studies on SNPs in genes regulating energy homeostasis and appetite—including *MC4R*, leptin (*LEP*), and *LEPR*—have shown that dietary interventions can have differential impacts on metabolic outcomes depending on the genotype [217,218,219,220]. A low-fat diet was associated with reduced LDL-c levels and improved triglyceride concentrations and HOMA-IR in obese Chinese men carrying the CC and CT genotypes of the *MC4R* SNP rs17782313 [217], whereas the C allele of this variant confers an increased risk of obesity and hyperglycemia [218]. *LEP* and *LEPR* are also associated with glucose and lipid homeostasis. Chinese individuals carrying the A allele of the *LEP* SNP rs7799039 exhibited higher fasting insulin concentrations and elevated HOMA-IR. In contrast, the *LEPR* SNPs rs1137101 and rs1805094 showed weak associations with leptin levels and markers of glucose and lipid metabolism in that same population [219]. However, the GG genotype of the *LEPR* SNP rs1137101 was associated with a significantly increased risk of T2D compared to the AA genotype in a study involving participants from southern India, as well as in individuals from various other geographic regions, including Malaysia, China (Han), Kuwait, Iran, Mongolia, Greece, and Saudi Arabia [220]. Taken together, these data may enhance our understanding of the role of genetic variants in the pathophysiology of T2D.

Genetic variants within the fatty acid desaturase 1 (*FADS1*) gene modulate individual responses to plant-based fats, particularly regarding the effect of nut consumption on circulating fatty acids. Specifically, *FADS1* SNPs influence the relationships between linoleic acid (LA) and ALA intake and nut consumption with plasma phospholipid fatty acid profiles and T2D risk. This was demonstrated in the large-scale EPIC-InterAct cohort study (7498 T2D cases and 10,087 subcohort participants) and in a randomized controlled clinical trial (492 participants in the MedDiet + Nuts intervention group; 436 controls). In both studies, nut intake exhibited a positive association with plasma LA levels and an inverse association with arachidonic acid, driven by the increased frequency of the minor C-allele of rs174547. While the interaction between nut consumption and T2D did not reach statistical significance, an inverse association was observed specifically in participants with the rs174547 CC genotype (HR: 0.73, 95% CI: 0.54–1.00) compared to CT (0.94, 0.81–1.10) or TT (0.90, 0.78–1.05) carriers in the EPIC-InterAct study [221].

Furthermore, a randomized controlled trial involving adult and elderly men homozygous for the SNP rs174550 in the *FADS1* gene (CC and TT genotypes) demonstrated a significant genotype-diet interaction regarding PUFA metabolism. Following an 8-week intervention with diets rich in linoleic acid (sunflower oil, 62–63% linoleic acid) or ALA (*Camelina sativa* oil, 30–35% ALA), these genotypes influenced plasma PUFA ratios, high-sensitivity C-reactive protein levels, and insulin secretion. At baseline, individuals with the TT genotype exhibited higher high-density lipoprotein cholesterol (HDL-c) concentrations and lower triglyceride levels compared to CC carriers. Following the intervention, total cholesterol and low-density lipoprotein cholesterol (LDL-c) concentrations decreased in the group consuming *Camelina sativa* oil, while ALA levels increased in both genotypes; however, EPA and docosapentaenoic acid (DPA) levels increased significantly only in carriers of the TT genotype [222].

Recent evidence from a systematic review reinforces the importance of gene–diet interactions in MetS. Across 40 observational studies, dietary fat intake was shown to interact with genetic variants involved in lipid metabolism, including *CAV-1* (rs3807992), *MC4R* (rs12970134), and *ACC2* (rs4766587), influencing individual susceptibility. Higher total and saturated fat intake were associated with increased risk among carriers of risk alleles, whereas PUFAs appeared to exert protective effects depending on genotype. Some studies also reported quantitative thresholds, including total fat intake above 64 g/day, saturated fat intake exceeding 25 g/day, and fat intake above 35% of total energy, associated with increased risk, while PUFA intake above 6% of energy may be protective. However, these findings remain heterogeneous and should be interpreted as study-specific rather than universal dietary recommendations [223].

Overweight and obese individuals from western Mexico were evaluated in a controlled clinical trial and separated into two groups: one receiving a standard diet, based on general guidelines for the treatment of dyslipidemia and obesity, and the other receiving a nutrigenetic diet, based on each individual’s genotype, to analyze associations between SNPs related to dyslipidemias in this population, such as *APOA1*, *APOC3*, *APOA5*, *APOE*, *ABCA1*, *CETP*, *LIPC*, *LPL*, *FABP2*, and *PPARG*, and the lipid and inflammatory profile. Both groups received a diet calculated between 25 and 30 kcal/kg of ideal weight with a caloric restriction of approximately 500 kcal, and differentiation of PUFA, monounsaturated fatty acids, and SFAs according to the diet pattern. A significant 20% reduction in triglycerides and pro-inflammatory cytokines IL-6 and TNF-α was observed in the nutrigenetic diet group, while the conventional diet intervention did not show beneficial changes in metabolic and inflammatory markers, although both groups demonstrated weight loss. Thus, this study indicates that the nutritional strategy for individuals with a high genetic risk for dyslipidemia should integrate genotypes to determine the adequate intake of mono- and PUFA [21].

Collectively, these findings support the notion that IR and cardiometabolic diseases arise from a complex interplay between genetic predisposition and environmental exposures, particularly dietary patterns. Nutrigenetic approaches provide a promising framework for precision nutrition by enabling the identification of individuals who may benefit from targeted dietary modifications, including adjustments in macronutrient composition, fatty acid profile, and the intake of specific bioactive compounds.

However, despite significant advances in GWAS and the identification of multiple risk loci, the translation of nutrigenetic evidence into clinical practice remains limited. This is largely due to the small effect sizes of individual variants, the complexity of gene–gene and gene–environment interactions, and the heterogeneity across populations, which collectively challenge the reproducibility and applicability of current findings.

Therefore, future research should prioritize the integration of multi-omics data, including genomics, metabolomics, and microbiome profiling, alongside the development of large-scale, ethnically diverse, and well-designed clinical trials. Additionally, the implementation of robust and clinically applicable algorithms capable of translating genetic information into actionable dietary recommendations will be essential. Advancing these approaches will be critical to fully realize the potential of precision nutrition in improving prevention and management strategies for obesity, IR, and cardiometabolic diseases.

### 4.3. Nutrimiromics: Dietary Regulation of MicroRNAs

Nutrimiromics refers to the field of nutritional genomics focused on studying how dietary components modulate gene expression through miRNA-mediated epigenetic mechanisms, as well as the impact of this interaction on the risk of developing chronic diseases [15,16]. Given the relevance of miRNAs in various biological processes, including those related to cardiometabolic risk factors, evidence from the literature has focused on understanding how nutrients, BCs, and dietary patterns are associated with these epigenetic mechanisms (Supplementary Table S2).

Within this context, the lipid composition of the diet serves as a critical modulator of both systemic inflammatory responses and miRNA-mediated regulatory networks [6,224]. In an acute postprandial context, it has been demonstrated that the consumption of a meal rich in SFAs in healthy women was able to trigger metabolic endotoxemia, characterized by a significant increase in plasma LPS concentrations, accompanied by peaks in triacylglycerols, TNF-α, and VCAM-1. This inflammatory state induced the differential expression of 33 plasma miRNAs at postprandial time points, with emphasis on miR-145-5p and miR-200, which were consistently modulated over time (Figure 2). These miRNAs are involved in the regulation of the NF-κB pathway; miR-145-5p suppresses pro-inflammatory cytokines via interaction with the CD40 receptor, whereas miR-200 negatively regulates the adaptor protein MyD88, which is essential for TLRs signaling. The authors suggest that these miRNAs appear to exert a “buffer-like action” in response to the inflammatory stress induced by SFAs and LPS via TLR4 [224].

Complementarily, lipid quality appears to reverse molecular profiles altered by diets rich in SFAs [225]. For example, the intake of omega-3 PUFAs, such as EPA and DHA, exerts cardioprotective effects through the TLR4/NF-κB pathway and reduces endothelial dysfunction [6]. In experimental models, diet-induced dyslipidemia was associated with hepatic overexpression of miR-122 and miR-33a, while DHA supplementation attenuated this response. This effect was accompanied by the repression of *Srebp2*, the host gene of miR-33a, and the disinhibition of its targets, such as ATP-binding cassette transporter A1 (*Abca1*), favoring cholesterol efflux and HDL synthesis. Furthermore, the expression of miR-33a in peripheral blood mononuclear cells (PBMCs) mirrored the hepatic pattern, suggesting its potential as a peripheral marker of lipid regulation [225].

Additionally, micronutrients and BCs in the diet have been associated with the differential expression of miRNAs involved in the regulation of redox homeostasis and inflammatory signaling [16,226]. Among the most studied BCs, polyphenols stand out for their ability to attenuate the systemic pro-inflammatory state through post-transcriptional regulation [16]. For example, polyphenols from extra virgin olive oil, specifically the secoiridoids oleocanthal and oleacein, have demonstrated anti-inflammatory effects in adipocyte models that mimic obesity. In human adipocytes, treatment with 25 µmol/L of these compounds for 6 h prevented the overexpression of miR-155-5p and miR-34a-5p and the reduction in let-7c-5p levels induced by TNF-α [227]. These alterations, observed both intracellularly and in exosomes, suggest modulation of intercellular communication in WAT [227].

Similarly, resveratrol, a phenolic compound that exerts anti-inflammatory effects through multiple mechanisms, including the downregulation of TLR4-NF-κB expression, also acts via molecular mechanisms mediated by miRNAs [16,24,174,175,228]. In experimental models of spinal cord injury and in LPS-stimulated PC-12 cells, resveratrol administration inhibited the inflammatory response by upregulating miR-132, an miRNA that mediates the blockade of p38MAPK and NF-κB pathways [228].

Regarding micronutrients, selenium stands out for composing antioxidant selenoproteins, such as glutathione peroxidases, which contribute to the reduction in ROS and protection against oxidative stress [229]. In vivo, selenium depletion induced the upregulation of five specific miRNAs in cardiac tissue: miR-374, miR-16, miR-199a-5p, miR-195, and miR-30e, which influence processes such as signal transduction and stress response [230].

Beyond the isolated action of nutrients, dietary patterns reflect the synergistic interaction between different dietary components. In this context, the MedDiet has been extensively studied, as it represents a complex dietary matrix rich in fiber, monounsaturated fatty acids, BCs, and micronutrients [191]. These components act in an integrated manner to regulate molecular mechanisms, including the modulation of gene expression through epigenetic modifications. Such regulation significantly influences metabolic homeostasis, the systemic inflammatory response, and the clinical progression of non-communicable diseases [144,231,232].

In the RESMENA randomized clinical trial, an eight-week hypocaloric intervention (30% energy restriction) based on the MedDiet standard altered miRNA expression in white blood cells of individuals with MetS. This clinical response was accompanied by the modulation of miRNAs in white blood cells, with increased expression of let-7b and reduced expression of miR-155. Notably, higher diet quality, assessed by the Healthy Eating Index, was positively associated with the expression of an miRNA signature composed of let-7b, miR-125b, miR-130a, miR-132-3p, and miR-422b. The reduction in SFA intake was considered a determinant of the increase in let-7b levels, an miRNA that regulates atherogenic and adipogenic processes, suggesting that improvements in dietary lipid quality induce epigenetic reprogramming in leukocytes, favoring an anti-inflammatory phenotype [231].

Concurrently, in the CENTRAL study population, the standard MedDiet supplemented with nuts (28 g/day) or a low-fat diet for 18 months induced dynamic changes in the serum miRNA profile that reflected the redistribution of body fat in individuals with abdominal obesity [232]. Post-intervention reductions in the expression of the miR-99/100 family (particularly miR-99a-5p and miR-100-5p) were associated with greater decreases in total and regional adiposity, including VAT, as well as reductions in intrahepatic and pancreatic fat [232]. Given the role of miR-100 in regulating adipogenesis and adipose tissue inflammation through targets such as *PPARG* and IGF-1R, its modulation suggests a systemic adaptation associated with the reduction in diabetogenic fat [232]. Taken together, these findings reinforce that qualitative changes in diet can influence the miRNA profile independently of the magnitude of weight loss.

In summary, this body of evidence positions diet as a promising epigenetic strategy, in which miRNA signatures act as mediators between dietary patterns and metabolic phenotypes. However, despite the promising findings reported to date, their interpretation should be approached with caution, given that some of the mechanistic evidence derives from in vitro [227] and animal models [225,228,230], which do not fully recapitulate the complexity of human physiology. Moreover, the experimental studies discussed evaluate isolated bioactive compounds, such as oleocanthal, oleacein, or resveratrol, whereas in humans, these compounds undergo biotransformation and metabolism before reaching target tissues, which may modify their biological activity [227,228]. Consequently, the molecular responses observed under controlled experimental conditions may not accurately reflect those elicited by habitual dietary intake in humans.

Additionally, the methodological heterogeneity among clinical trials, including differences in dietary intervention protocols, participant characteristics, quantification techniques, and the biological matrices analyzed, hinders direct comparisons across studies and contributes to inconsistencies in the reported findings [224,231,232].

Finally, miRNA expression can be specific to particular tissues and cell types [233,234]. Therefore, circulating miRNAs may not accurately reflect the molecular events occurring in metabolically active tissues, such as the liver or adipose tissue [234]; consequently, this biological compartmentalization should be considered when interpreting circulating miRNAs as biomarkers or potential mediators of dietary effects. Future studies should prioritize conducting randomized clinical trials in humans and integrating analyses of circulating and tissue-specific miRNAs to better understand whether these molecules primarily act as biomarkers of metabolic adaptation or as functional mediators of dietary responses.

### 4.4. Epigenetics: Nutrition and DNA Methylation

**Figure 4:**
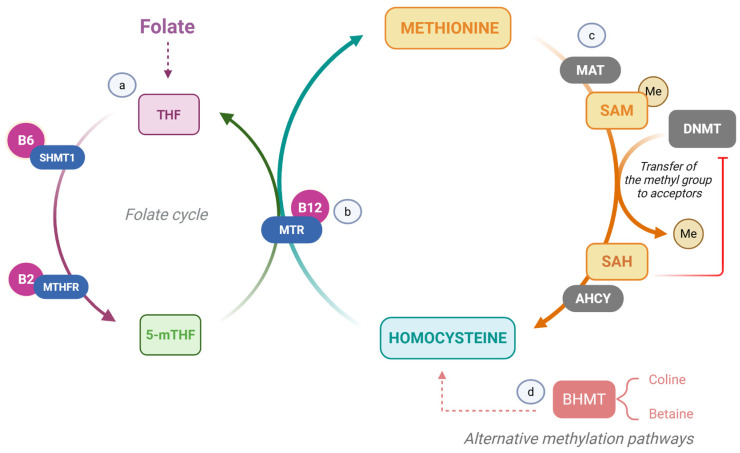
Overview of the one-carbon metabolic pathway. Dietary folate is converted to its active form, tetrahydrofolate (THF), which enters the folate cycle. (**a**) Within this cycle, THF is converted to 5,10-methylenetetrahydrofolate by serine hydroxymethyltransferase 1 (SHMT1), a vitamin B6–dependent enzyme and subsequently reduced to 5-methyltetrahydrofolate (5-mTHF) by methylenetetrahydrofolate reductase (MTHFR), which is dependent on vitamin B2. (**b**) 5-mTHF donates a methyl group to homocysteine in a reaction catalyzed by methionine synthase (MTR), a vitamin B12–dependent enzyme, forming methionine and regenerating THF, which can be recycled within the folate cycle. (**c**) Methionine is converted to S-adenosylmethionine (SAM) by methionine adenosyltransferase (MAT). SAM acts as the universal methyl donor in reactions catalyzed by DNA methyltransferases (DNMT), generating S-adenosylhomocysteine (SAH), which inhibits the action of DNMT. SAH is subsequently hydrolyzed to homocysteine by S-adenosylhomocysteine hydrolase (AHCY), thereby completing the cycle. (**d**) Homocysteine can also be remethylated through folate-independent alternative pathways, in which choline and betaine serve as methyl donors in a reaction catalyzed by betaine–homocysteine methyltransferase (BHMT). **Symbol:** ⊣, inhibition; Source: Created by the authors using BioRender. Created in BioRender. Corsi, G. (2026) https://BioRender.com/vgq79oc.

As discussed previously, SAM, synthesized from methionine, represents the primary methyl donor for methylation reactions. Following the transfer of its methyl group to specific acceptors, SAM is converted into S-adenosylhomocysteine (SAH), a potent inhibitor of DNA methyltransferases. In this context, the availability of dietary substrates and cofactors plays a central role in sustaining one-carbon metabolism. Methionine, an essential amino acid, must be obtained from the diet, as must folate, whose active form—tetrahydrofolate (THF)—enters the folate cycle and supports the remethylation of homocysteine. The conversion of THF to 5-methyltetrahydrofolate (5-mTHF) depends on enzymes requiring vitamin cofactors, including vitamins B6 and B2, while the transfer of the methyl group from 5-mTHF to homocysteine is catalyzed by the vitamin B12–dependent enzyme methionine synthase, leading to the regeneration of methionine and the continuity of the methionine cycle [235]. Additionally, other dietary compounds such as choline and betaine contribute to alternative folate-independent remethylation pathways (Figure 4). Diet, therefore, acts not only as a source of substrates but also as a modulator of the metabolic flux of this pathway, such that the quantity of nutrients available for the reactions may influence the DNA methylation pool across the entire genome [25,235].

Diet influences DNA methylation as early as the prenatal period, with growing evidence supporting the idea that methylome modulation resulting from parental dietary exposures is transmitted to offspring, influencing the risk of non-communicable diseases [236,237,238]. Postnatal nutrition, particularly early in life, may affect differential DNA methylation and impact disease risk [238,239,240]. The consumption of animal and plant proteins may influence the childhood methylome in distinct ways. Plant protein intake has been associated with CpG sites of the *C1orf159* and *MBP* genes in early childhood, whereas animal protein intake has only been relevant in late childhood, being associated with the methylation of the *HOXB9* and *MARCHF1* genes, the latter of which plays a role in glycolipid homeostasis [240], highlighting the relevance of life cycle factors in this modulation.

Also, the methylation of genes related to obesity (*NRF1*, *FTO*, and *LEPR*) responds differently to the intake of methyl-donor nutrients depending on the child’s nutritional status, since dietary folate intake in children with normal weight has been associated with *NRF1* methylation, whereas the same was not observed in children with overweight or obesity. In the same study, riboflavin intake was positively associated with methylation of the *NRF1* and *FTO* genes only in eutrophic children, although both riboflavin intake and methylation of the respective genes were higher in children with overweight/obesity, suggesting that sensitivity to dietary modulation may be more pronounced when a homeostatic state still predominates [239]. This characteristic, however, appears not to be limited to children. Li et al. [241] reported that adults with overweight or obesity who had higher baseline methylation in *TXNIP* showed greater reductions in insulinemia and HOMA-IR after a weight-loss diet, particularly with moderate protein intake, suggesting that changes in protein content tend to be more relevant for individuals who still exhibit higher methylation of this gene. Nevertheless, differences in study protocols and population characteristics warrant caution when extrapolating these findings.

In this context, the epigenetic modulation promoted by diet appears to depend largely on the baseline physiometabolic state. Increased body adiposity can compromise pathways related to the utilization of micronutrients involved in DNA methylation, directly impacting the methylome. Folic acid, for instance, appears to exhibit impaired metabolism in obesity [239,242,243]. Individuals with obesity frequently present lower serum folate concentrations compared with eutrophic individuals, even under supplementation protocols. Evidence suggests that conventional folic acid recommendations may be insufficient to adequately meet the metabolic demands associated with obesity. Although the underlying mechanisms have not yet been fully elucidated, several pathophysiological hypotheses have been proposed to explain this association. Among them, increased activity of cytochrome P450 enzymes observed in obesity has been highlighted, particularly CYP2E1, which has been associated with oxidative folate degradation. In addition, elevated concentrations of MeFox—the primary oxidation product of 5-mTHF—have also been reported in this population, suggesting greater folate instability and oxidative turnover [243]. Collectively, these findings indicate that the metabolic dysfunction and oxidative stress may impair folic acid utilization, thereby contributing to increased physiological requirements for this nutrient.

Regarding dietary patterns, a randomized clinical trial demonstrated that adherence to a polyphenol-rich MedDiet may be superior to both a standard healthy diet and the conventional MedDiet in promoting epigenetic changes in individuals with dyslipidemia or abdominal obesity. This intervention was able to induce more pronounced reductions in deep SAT, as well as promote greater increases in circulating concentrations of vitamin B12 and folate, which may explain the higher number of differentially methylated sites in this group. However, in the same study, changes in the methylation and transcription of genes encoding key proteins involved in epigenetic regulation, such as *KDM2B* and *KDM5B* (lysine demethylases) and *SETD1A* (SET domain-containing 1A histone lysine methyltransferase), were observed, suggesting that this modulation is not limited to the provision of key nutrients involved in one-carbon metabolism, but rather has a broader effect—possibly associated with polyphenols—capable of modulating the enzymatic machinery responsible for regulating the human epigenome [244]. The consumption of nuts and legumes—foods rich in flavonoids and other polyphenols—is one of the pillars of the MedDiet. Evidence suggests that these foods, in addition to providing methyl donors, can interact with DNA methyltransferases involved in several pathways related to metabolic dysfunction and inflammation, which highlights the potential of polyphenols in epigenetic regulation [245].

The MedDiet combined with nut supplementation also showed more relevant results in another randomized double-blind clinical trial conducted in older adults. Enrichment with nuts promoted increased methylation in peripheral white blood cells at the CpG site cg01081346, whose genes correspond to *CHKB-CPT1B/CPT1B*, which encode enzymes involved in fatty acid oxidation. This site was also correlated with the intake of PUFA, which constitute the main source of fats in the MedDiet, further highlighting the relevance of fat type in epigenetic modulation [246]. In parallel, the Korean diet, characterized by the predominant consumption of foods such as grains, fruits, vegetables, legumes, fish, nuts, and seafood, proved to be superior to a Westernized diet—with higher intake of red meat and refined grains—in its ability to induce DNA methylation in PBMCs, as well as to improve the lipid profile. This modulation likely reflects the wide availability of B-complex vitamins in this dietary pattern [247].

Other dietary micronutrients may likewise affect DNA methylation. Daily zinc intake, for example, has been positively associated with methylation in the Italian population [248]. Furthermore, in a meta-analysis of EWAS, 4656 and 160 differentially methylated CpG sites were identified in response to vitamin C and vitamin E intake, respectively. In addition to its antioxidant action, vitamin C acts as a cofactor for TET dioxygenases involved in active DNA demethylation, consistent with the predominant hypomethylation observed in the study. Vitamin E exerts a more indirect effect, acting as an antioxidant and in the regeneration of vitamin C, which may explain the smaller number of methylated sites observed [249].

However, it should be noted that findings regarding the direction of methylation remain controversial. da Mota et al. [250], in a systematic review and meta-analysis aimed at evaluating the impact of methyl-donor nutrient supplementation on DNA methylation, observed that folic acid supplementation does not necessarily result in hypermethylation. The results of the meta-analysis conducted with human studies indicated no significant changes associated with folic acid supplementation, whereas the meta-analysis of animal models identified a significant dose-dependent association, in which higher doses were positively associated with methylation. The authors noted that the studies included in the review were highly heterogeneous, differing in population characteristics, loci evaluated, analytical methods, the types of biological samples analyzed, as well as in supplementation protocols, which included folic acid alone and/or in combination with other nutrients. Similarly, as highlighted in the present review, current evidence supports a role for diet in modulating DNA methylation. However, substantial heterogeneity remains in study protocols that may limit the generalizability of these findings.

## 5. Conclusions and Future Directions

Obesity associated with the phenotype of excess visceral adiposity, IR, and chronic low-grade systemic inflammation constitutes a significant risk factor for the development of non-communicable diseases. Metabolic dysfunction related to obesity results not only from a positive energy balance but also from a complex interaction between genetic predisposition, diet, and epigenetic mechanisms. Collectively, the evidence reviewed indicates that genetic variants, particularly SNPs in genes such as *TCF7L2*, *FTO*, *PNPLA3*, and *IRS1*, contribute to susceptibility to cardiometabolic disorders. Complementarily, epigenetic regulatory layers, including miRNAs and DNA methylation, act as dynamic mediators at the interface between nutritional and metabolic stimuli, modulating gene expression in both specific tissues and at the systemic level, facilitating interorgan communication and impacting central metabolic pathways in the pathogenesis of chronic diseases such as T2D and CVD.

In this context, healthy dietary patterns, as well as specific nutrients and BCs, have the potential to modulate inflammatory pathways—such as TLR4/NF-κB signaling—and optimize glycemic homeostasis, while also influencing the epigenetic regulatory mechanisms involved in these pathways. However, the clinical translation of these findings still faces important limitations, including the small effect sizes of individual genetic variants, the methodological heterogeneity of nutritional intervention studies, and the need for functional validation of epigenetic mechanisms, particularly those involving miRNAs and DNA methylation. Furthermore, this review has the inherent limitation of its narrative design, as it is based on a qualitative synthesis of the literature and is therefore subject to methodological heterogeneity and potential selection bias among the included studies. Overall, this review highlights the potential of integrative multi-omics approaches to advance our understanding of the complex interactions among genetic predisposition, epigenetic mechanisms, and dietary exposures. Continued methodological advances, functional validation, and clinical implementation studies will be essential to determine how these approaches can contribute to future precision nutrition strategies for cardiometabolic diseases.

## Data Availability

No new data were created or analyzed in this study. Data sharing is not applicable to this article.

## Supplementary Materials

The following supporting information can be downloaded at: https://www.mdpi.com/article/10.3390/metabo16070501/s1, Table S1: SNPs related to insulin resistance, inflammation, and obesity; Table S2: Dietary effects of miRNA expression: evidence from experimental and clinical studies. References [251,252,253,254,255,256,257,258,259,260,261,262] are cited in the Supplementary Materials.

## Abbreviations

The following abbreviations are used in this manuscript:

5-mTHF	5-methyltetrahydrofolate
ABCA1	ATP-Binding Cassette Subfamily A Member 1
ABCG1	ATP binding cassette subfamily G member 1
ACC	Acetyl-CoA carboxylase
ADIPOQ	Adiponectin, C1Q and collagen domain containing
AHCY	S-adenosylhomocysteine hydrolase
ALA	Alpha-linolenic acid
AMPK	AMP-Activated Protein Kinase
APOA1, APOC3, APOA5, APOE	Apolipoproteins A1, C3, A5, and E
AP-1	Activator protein-1
ASC	Apoptosis-associated speck-like protein
BC	Bioactive compounds
BHMT	Betaine–homocysteine methyltransferase
BMI	Body mass index
C/EBPβ	CCAAT/Enhancer-Binding Protein Beta
CCL	C-C motif chemokine ligand
CD14, CD40	Cluster of differentiation 14 and 40
CETP	Cholesteryl ester transfer protein
ChREBP	Carbohydrate response element-binding protein
COX-2	Cyclooxygenase-2
CPT1A	Carnitine Palmitoyltransferase 1A
C1orf159	Chromosome 1 open reading frame 159
CVD	Cardiovascular diseases
DAG	Diacylglycerol
DAMPs	Damage-associated molecular patterns
DGCR8	DiGeorge Syndrome Critical Region 8
DHA	Docosahexaenoic acid
DHCR24	24-dehydrocholesterol reductase
DICER	RNase III Endonuclease Dicer
DNA	Deoxyribonucleic Acid
DNMT	DNA methyltransferases
DROSHA	RNase III Endonuclease Drosha
EPA	Eicosapentaenoic acid
FFA	Free fatty acid
FFAR	Free fatty acid receptors
FGF21	Fibroblast growth factor 21
FIH	Factor inhibiting HIF
FTO	Fat mass and obesity associated
GIPR	Gastric Inhibitory Polypeptide Receptor
GLUT4	Glucose transporter type 4
GPCR	G-protein coupled receptor
GWAS	Genome-wide association studies
HDL-c	High-density lipoprotein cholesterol
HIF	Hypoxia-inducible factors
HOMA-IR	Homeostatic Model Assessment of Insulin Resistance
HOXB9	Homeobox B9
ICAM-1	Intercellular adhesion molecule-1
IGF-1R	Insulin-like Growth Factor 1 Receptor
IκB	Inhibitor of κB
IKK	IκB kinase
IL	Interleukin
iNOS	Inducible nitric oxide synthase
IR	Insulin Resistance
IRS	Insulin receptor substrates
IRAK	Interleukin-1 Receptor-Associated Kinase
JNK	c-Jun N-terminal kinase
JUN	Jun proto-oncogene, AP-1 transcription factor subunit
KDM2B, KDM5B	Lysine Demethylase 2B and Lysine Demethylase 5B
KLF4	Krüppel-Like Factor 4
LBP	Lipopolysaccharide-binding protein
LEPR	Leptin receptor
LKB1	Liver Kinase B1
LPS	Lipopolysaccharide
MARCHF1	Membrane associated ring-CH-type finger 1
MAPK	Mitogen-activated protein kinase
MAPKAP1	MAPK Associated Protein 1
MASLD	Metabolic steatotic liver disease
MedDiet	Mediterranean diet
MAT	Methionine adenosyltransferase
MBP	Myelin basic protein
MetS	Metabolic syndrome
MD-2	Myeloid differentiation factor 2
MiRNAs	MicroRNAs
mRNA	Messenger RNA
mTORC2	Mechanistic target of rapamycin complex 2
MTHFR	Methylenetetrahydrofolate reductase
MTR	Methionine synthase
MyD88	Myeloid differentiation primary response 88
NF-κB	Nuclear Factor Kappa B
NLRP3	NOD-like receptor protein 3
NRF1	Nuclear respiratory factor 1
PAMPs	Pathogen-associated molecular patterns
PBMCs	Peripheral Blood Mononuclear Cells
PDK1	Pyruvate dehydrogenase kinase 1
PHD	Prolyl hydroxylase domain enzyme
PI3K	Phosphoinositide 3-Kinase
PIP2	Phosphatidylinositol-4,5-bisphosphate
PIP3	Phosphatidylinositol-3,4,5-trisphosphate
PKB	Protein kinase B
PNPLA3	Patatin-like phospholipase domain-containing protein 3
PPARA, PPARD, PPARG	Peroxisome Proliferator-Activated Receptor Alpha, Delta and Gamma
PPARGC1A	PPARG coactivator 1 alpha
PRDM16	PR domain containing 16
PRKAB1	Protein Kinase AMP-Activated Non-Catalytic Subunit Beta 1
PRRs	Pattern recognition receptors
PUFA	Polyunsaturated fatty acids
PTPRN2	Protein Tyrosine Phosphatase Receptor Type N2
p38MAPK	p38 Mitogen-Activated Protein Kinase
RAN-GTP	Ras-related Nuclear Protein bound to GTP
RISC	RNA-Induced Silencing Complex
ROS	Reactive oxygen species
SAM	S-adenosylmethionine
SAT	Subcutaneous adipose tissue
SFAs	Saturated fatty acids
SETD1A	SET domain containing 1A, histone lysine methyltransferase
SFRP4	Secreted Frizzled-Related Protein 4
SHIP1	Src homology 2 domain-containing inositol 5-phosphatase 1
SHMT1	Serine hydroxymethyltransferase 1
SREBF1	Sterol Regulatory Element Binding Transcription Factor 1
SNPs	Single nucleotide polymorphisms
SOCS	Suppressor of cytokine signaling
SOCS1	Suppressor of cytokine signaling 1
SREBP	Sterol Regulatory Element-Binding Protein
T2D	Type 2 Diabetes
TAB	TAK-binding proteins
TAK1	Transforming growth factor-β–activated kinase 1
TET	Ten-eleven translocation
TCF7L2	Transcription factor 7-like 2
TGF-β	Transforming growth factor-β
TIR	Toll/Interleukin-1 Receptor
TIRAP	TIR domain-containing adaptor protein
TNF-α	Tumor necrosis factor alpha
TLR	Toll Like Receptor
TRAF	Tumor Necrosis Factor Receptor Associated Factor
TXNIP	Thioredoxin Interacting Protein
THF	Tetrahydrofolate
UTR	Untranslated Region
VAT	Visceral adipose tissue
VCAM-1	Vascular cell adhesion molecule-1
WAT	White adipose tissue
Wnt/β-catenin	Wingless/Integrated–beta-catenin signaling pathway.
ZnT8	Zinc ions
