# Supplementary material for: Molecular Mechanisms Associated with Metabolic Dysfunction: Contributions of Nutritional Genomics
Source: Metabolites. 2026 Jul 16;16(7):501. doi: 10.3390/metabo16070501 (PMC13413746; doi:10.3390/metabo16070501)
Supplement: Supplementary file 1 [file metabolites-16-00501-s001.zip › metabolites-4388980-supplementary.pdf]

Supplementary Materials

Table S1. *SNPs related to insulin resistance, inflammation, and obesity.*

Gene	Gene function	SNP	Risk allele	Dietary interaction	Association with IR, inflammation, and obesity	Population/Sample	Author (year)
FTO ("alpha-ketoglutarate–dependent dioxygenase")	Energy metabolism and regulation of appetite and satiety	rs9939609 (T > A)	T	No significant differences in energy and nutrient intake were observed by genotype.	TT genotype carriers showed higher waist-to-height ratio, insulin, triglycerides, VLDL-c, and higher odds of IR, hypertriglyceridemia, and hyperglycemia.	West Mexican	[95]
MC4R ("melanocortin-4 receptor")	Energy homeostasis	rs17782313 (T > C)	C	–	Associated with higher risk of obesity and hyperglycemia. Elevated BMI: European Caucasian and East Asian populations; Elevated waist circumference and glycemia: European Caucasian; Elevated waist-to-hip ratio: East Asian.	European Caucasian and East Asian populations.	[218]
		rs17782313 (T > C)	C	Hypolipidic diet	Associated with reduced LDL-c, triglycerides, and HOMA-IR in men (CC and TC genotypes).	Chinese	[217]

Gene	Gene function	SNP	Risk allele	Dietary interaction	Association with IR, inflammation, and obesity	Population/Sample	Author (year)
TMEM18 (“transmembrane protein 18”)	Insulin and glucagon signaling	rs6548238 (T > C)	C	High-fat diet	Associated with obesity-related indicators; loci near the SNP may simultaneously increase susceptibility to T2D, associated with obesity (CC and TC genotypes).	Chinese	[251]
				–		Mexican	[252]
PNPLA3 (“patatin-like phospholipase domain-containing protein 3” or “adiponutrin”)	Triglyceride hydrolysis in adipocytes; energy metabolism and lipid homeostasis	rs738409 (C > G)	G	–	Individuals with the GG genotype show higher probability of developing MASLD.	Asian	[212]
				–	Associated with IR and greater hepatic susceptibility to metabolic dysfunctions, such as obesity and T2D. Both carriers of the allelic variant and non-carriers exhibited IR.	Hispanic; Caucasian; African American	[213]
SLC30A8 (“solute carrier family 30 member 8”)	Encodes a protein that ZnT8 to insulin secretory granules in pancreatic β-cells, essential for insulin storage	rs13266634 (C > T)	T	High-fat diet with mixed macronutrients	Associated with reduced triglyceride clearance in adults with BMI ≥ 30, leading to lipid accumulation and future metabolic dysfunctions such as IR.	American (USA)	[253]

Gene	Gene function	SNP	Risk allele	Dietary interaction	Association with IR, inflammation, and obesity	Population/Sample	Author (year)
			C	–	Associated with a protective role against susceptibility to T2D in individuals with the TT genotype.	Bangladeshi	[93]
IRS1 ("insulin receptor substrate 1")	Encodes a protein phosphorylated by the insulin receptor tyrosine kinase (insulin signaling)	rs1801278 (Gly972Arg) (C > T)	T	–	Associated with risk of gestational diabetes mellitus in the recessive model (TT).	Asian; Caucasian	[210]
		rs2943641 (T > C)	C	Meals containing beef or lamb compared with grain-based meals.	Association with significantly higher fasting insulin levels in individuals with CT and CC genotypes.	Multiethnic, predominantly European, Indian, Chinese, and Korean (New Zealand).	[20]
		rs7578326 (A > G)	A	Diets low in monounsaturated fatty acids, low total fat intake, or low fat-to-carbohydrate ratio.	Associated with lower risk of MetS in carriers of the G allele.	European; Puerto Rican	[211]
TRAIL (*TNFSF10*) ("TNF-related apoptosis-inducing ligand")	Encodes a cytokine belonging to the TNF family that selectively induces	rs767450259 (T > C)	C	–	The T allele may have a protective role against obesity.	Turkish	[254]

Gene	Gene function	SNP	Risk allele	Dietary interaction	Association with IR, inflammation, and obesity	Population/Sample	Author (year)
	apoptosis of transformed/tumor cells via the extrinsic pathway without damaging normal cells. It is involved in the pro-inflammatory process of adipose tissue.	rs369143448 (G > A)	A	–	The G allele may have a protective role against obesity.		
		rs750556128 (A > G)	G	–	The A allele may increase (≈1.3-fold) the risk of T2D in individuals with obesity.		
LEP ("leptin")	Regulation of energy homeostasis.	rs7799039 (G > A)	A	–	Associated with increased risk of IR and lower leptin concentrations.	Caucasian; Latin American; Asian; African.	[219]
LEPR ("leptin receptor")"	Regulation of lipid metabolism	rs 1137101 (A > G)	G	–	Associated with greater susceptibility to the development of T2D.	Indian	[220]

Gene	Gene function	SNP	Risk allele	Dietary interaction	Association with IR, inflammation, and obesity	Population/Sample	Author (year)
ADIPOQ ("adiponectin")	Regulation of glucose concentrations and fatty acid degradation; involved in metabolic and hormonal processes.	rs266729 (C > G); rs3774261 (A > G); rs2241766 (T > G).	G	–	Significant association with risk of MASLD in the recessive model.	Asian; Chinese; Caucasian.	[255]
		rs2241766 (T > G)	G	–	Associated with risk of T2D and other complications, such as MetS and gestational diabetes.	Asian; Caucasian	[256]
		rs1501299 (G > T)	T		Related to metabolic outcomes and insulin sensitivity; significant association with risk of diabetic retinopathy.		
TCF7L2 ("transcription factor 7-like 2")	Key role in the Wnt signaling pathway (T-cell–specific, HMG box).	rs7903146 (C > T)	T	–	Associated with transcription factors that regulate insulin production and secretion.	Asian; Hispanic/Latina; Caucasian	[87]
					Associated with significantly increased risk of developing prediabetes.	Kazakh	[97]

Gene	Gene function	SNP	Risk allele	Dietary interaction	Association with IR, inflammation, and obesity	Population/Sample	Author (year)
					Associated with increased risk of T2D; potential genetic risk marker.	Irish; Danish; American (USA); French; British European; Indian; Japanese	[89]
					Associated with increased risk of T2D and higher fasting glucose and high-sensitivity C-reactive protein concentrations.	Chinese	[90]
PPARG ("peroxisome proliferator-activated receptor gamma")	Regulator of adipocyte differentiation	rs1801282	G	–	Associated with metabolic alterations, adipogenesis, and risk of prediabetes.	Kazakh	[97]
IL6 ("interleukin 6")	Acts in inflammation and maturation of B lymphocytes	rs1800795 (C > G)	C	–	Associated with obesity and low-grade inflammation (meta-analysis).	French-Canadian; Caucasian; Indian	[98]
					Related to obesity risk in population studies.	Turkish	[99]
		rs1800796 (G > C)	C	-	Significant association with increased risk of obesity.	Turkish	[99]

Gene	Gene function	SNP	Risk allele	Dietary interaction	Association with IR, inflammation, and obesity	Population/Sample	Author (year)
TNF ("tumor necrosis factor")	Encodes a multifunctional pro-inflammatory cytokine involved in a wide range of biological processes, including lipid metabolism and apoptosis of pancreatic β-cells	rs1800629 (G > A)	A	–	Associated with increased risk of developing T2D.	Chinese (Han)	[257]
		rs1799964 (T > C)	C	–	CC genotype associated with higher glycated hemoglobin (HbA1c) levels in non-diabetic individuals.		
		rs1799724 (C > T)	T	–	Associated with increased risk of T1D.	Chinese; East Asian; Caucasian; Middle Eastern	[258]
		rs1800629 (G > A)	A	–	Related to TNF-α expression and metabolic inflammation.	Egyptian	[259]
			A	–	Associated with IR markers and susceptibility to T2D.	Pakistani (Punjab)	[100]
		rs361525 (G > A)	A	–	Associated with susceptibility to T2D.		
		rs1800629 (G > A)	A	Dietary antioxidants	Associated with greater waist circumference, higher fat-to-lean mass ratio, and, in men, higher insulin concentrations than in women. May interact with	Spanish	[22]

Gene	Gene function	SNP	Risk allele	Dietary interaction	Association with IR, inflammation, and obesity	Population/Sample	Author (year)
					dietary factors in the modulation of IR.		
TLR4 (“Toll-like receptor 4”)	Pathogen recognition and activation of innate immunity. Through signaling pathways, insulin action is impaired, leading to IR in tissues such as liver, muscle, and adipose tissue.	rs4986790 (A > G)	G	–	Related to response to LPS, obesity, and metabolic inflammation.	Mexican	[104]
					Associated with phenotypic characteristics such as abdominal obesity.	Saudi	[103]
		rs4986791 (C > T)	T	–	Associated with obesity and metabolic alterations.	Mexican	[104]
		rs5030717 (A > G)	A	–	SNP originally related to elderly individuals showed significant association with obesity parameters (visceral fat, body fat percentage, and increased waist circumference).	Italian	[260]

Gene	Gene function	SNP	Risk allele	Dietary interaction	Association with IR, inflammation, and obesity	Population/Sample	Author (year)
			G	–	Modulates the inflammatory response via the TLR4/NF-κB pathway. Associated as a potential predictive biomarker for multiple complications, such as nephropathy, hypertension, and dyslipidemia in T2D.	Indian	[261]
IL10 ("interleukin 10")	Anti-inflammatory cytokine with immunoregulatory effects; inhibits pro-inflammatory cytokines, modulates NF-κB and JAK-STAT activation, and influences immune cell function, including B cells	rs1800872 (T > G)	T	-	Associated with susceptibility to dyslipidemia in individuals (TT genotype) with T2D and hypertension.	Chinese	[262]

Abbreviations: BMI, body mass index; HbA1c, glycated hemoglobin; HDL-c, high-density lipoprotein cholesterol; HOMA-IR, homeostasis model assessment of insulin resistance; hs-CRP, high-sensitivity C-reactive protein; IR, insulin resistance; JAK-STAT, Janus kinase/signal transducer and activator of transcription pathway; LDL-c, low-density lipoprotein cholesterol; LPS, lipopolysaccharide; MASLD, metabolic dysfunction–associated steatotic liver disease; MetS, metabolic syndrome; NF-κB, nuclear factor kappa B; SFA, saturated fatty acids; SNP, single-nucleotide polymorphism; T1D, type 1 diabetes; T2D, type 2 diabetes; TG, triglycerides; ZnT8, zinc transporter 8.

Table S2. *Dietary effects of miRNA expression: evidence from experimental and clinical studies.*

Dietary Exposure	Biological Matrix	miRNA Regulation	Targets	Key Findings	Reference
High-fat high-saturated meal	Plasma	↑ miR-145<br>↑ miR-200	*CD40, TIRAP, IFN-β, MyD88, NF-kB* signaling	SFA intake induces metabolic endotoxemia and postprandial inflammation. The increase in miR-145 and miR-200 suggests a buffer-like action to mitigate the inflammatory response.	[224]
Docosahexaenoic Acid (DHA)	Liver and PBMCs	↓ miR-33a<br>↓ miR-122	pparβ/δ, cpt1a, abca1	DHA acts by repressing the host gene *Srebp2*, promoting HDL synthesis and cholesterol efflux via ABCA1.	[225]
Resveratrol	Rat spinal cord and PC-12 neuronal cells	↑ miR-132	p38MAPK, NF-kB signaling	Resveratrol increases the expression of miR-132, which inhibits the NF-κB and p38MAPK pathways, reducing apoptosis and inflammation.	[228]
Secoiridoids oleocanthal and oleacein	Adipocytes and exosomes	↓ miR-155-5p,<br>↓ miR-34a-5p,<br>↑ let-7c-5p	p65	Oleocanthal and oleacein attenuate inflammation and adipocyte dysfunction by inhibiting *NF-κB* activation and regulating specific miRNAs both intracellularly and in exosomes.	[227]
Selenium	Cardiac tissue and plasma	↑ miR-374<br>↑ miR-16<br>↑ miR-199a	Wnt/β-catenin signaling	Selenium deficiency is a causal factor in heart failure. This process is mediated by activation of the Wnt/β-catenin	[230]

Dietary Exposure	Biological Matrix	miRNA Regulation	Targets	Key Findings	Reference
		↑ miR-195<br>↑ miR-30e<br>↓ miR-3571<br>↓ miR-675<br>↓ miR-450a		pathway and is associated with miRNA regulation.	
Mediterranean Diet	White blood cells	↓ miR-155<br>↑ miR-let-7b	*CD40, SHIP1, C/EBPb*	MedDiet modulates miR-155-3p and let-7b expression in leukocytes. These changes were driven by overall diet quality (Healthy Eating Index) and reduced saturated fat intake, independently of weight loss.	[231]
Low-fat diet or Mediterranean Low-Carbohydrate Diet: associated or not with physical activity.	Serum	↓ miR-99a<br>↓ miR-100<br>↓ miR-99b	*PPAR, FGF21, IGF1R*	The reduction in circulating miRNAs (miR-99a, miR-99b, and miR-100) is associated with improved ectopic visceral fat distribution in patients with abdominal obesity.	[232]

Direction of regulation (↑ upregulation; ↓ downregulation). Abbreviations: ABCA1, ATP-binding cassette transporter A1; C/EBPβ, CCAAT/enhancer-binding protein beta; CD40, cluster of differentiation 40; CPT1A, carnitine palmitoyltransferase 1A; DHA, docosahexaenoic acid; FGF21, fibroblast growth factor 21; IFN-β, interferon beta; IGF1R, insulin-like growth factor 1 receptor; MyD88, myeloid differentiation primary response 88; NF-κB, nuclear factor kappa B; p38 MAPK, p38 mitogen-activated protein kinase; PBMCs, peripheral blood mononuclear cells; p65, nuclear factor kappa B subunit p65; PPAR, peroxisome proliferator-activated receptor; PPARβ/δ, peroxisome proliferator-activated receptor beta/delta; SFA, saturated fatty acids; SHIP1, Src homology 2 domain-containing inositol 5-phosphatase 1; SREBP2, sterol regulatory element-binding protein 2; TIRAP, Toll/interleukin-1 receptor (TIR) domain-containing adaptor protein; Wnt/β-catenin, Wingless/Integrated–beta-catenin signaling pathway.
